# Effect of Diet and Dietary Supplements on Gout-Related Outcomes: A Systematic Review of Randomised Controlled Trials

**DOI:** 10.31138/mjr.010725.era

**Published:** 2025-12-31

**Authors:** Eleni C. Pardali, Arriana Gkouvi, Kalliopi Nasiou, Christos Cholevas, Theodoros N. Sergentanis, Eleni Kornarou, Ioannis K. Sarris, Dimitrios G. Goulis, Dimitrios P. Bogdanos, Tonia Vassilakou, Maria G. Grammatikopoulou

**Affiliations:** 1 Immunonutrition Unit, Department of Rheumatology and Clinical Immunology, Faculty of Medicine, School of Health Sciences, University of Thessaly, Larissa, Greece;; 2 Department of Public Health Policy, School of Public Health, University of West Attica, Athens, Greece;; 3 Laboratory of Pharmaceutical Technology, Division of Pharmaceutical Technology, School of Pharmacy, Faculty of Health Sciences, Aristotle University of Thessaloniki, Thessaloniki, Greece;; 4 Academic Orthopaedic Department, Papageorgiou General Hospital, Aristotle University Medical School, Thessaloniki, Hellas, Greece;; 5 Unit of Reproductive Endocrinology, 1^st^ Department of Obstetrics and Gynecology, Papageorgiou General Hospital, Medical School, Aristotle University of Thessaloniki, Thessaloniki, Greece

**Keywords:** gouty arthritis, dietary supplements, diet therapy, nutrition therapy, uric arthritis

## Abstract

**Objective:**

Gout is an inflammatory arthritis caused by elevated serum uric acid (SUA) concentrations, leading to monosodium urate crystal deposition in the joints and clinical symptoms. Various dietary supplements and dietary patterns have been explored as potential strategies to reduce SUA concentrations and alleviate gout manifestations.

**Methods:**

A systematic review was conducted across three major databases (PubMed, EBSCO, and clinicaltrials.gov), to identify randomised controlled trials evaluating the effects of dietary supplements and dietary patterns on gout-related outcomes. Although a meta-analysis was not performed, 27 studies met the inclusion criteria and were synthesised narratively.

**Results:**

Most studies investigated outcomes such as SUA concentrations, frequency of gout flares, joint pain, swelling, tenderness, renal function, cardiometabolic parameters, anthropometric measures, and quality of life. The assessed supplements included cherry juice or extract, n-3 fatty acids, vitamin C, and various traditional Chinese medicine (TCM) formulations, such as Rebixiao granules, compound Tufuling oral liquid, compound Qingbi granules, Yellow-Dragon Wonderful-Seed, modified Simiao Tang, Chuanhu, Weicao, and the Simiao Pill. The findings were mixed and often contradictory, with some studies reporting improvements in SUA levels and gout flare frequency, while others suggesting no effect. Additionally, many studies reported a range of adverse events.

**Conclusion:**

Current evidence on the effectiveness of dietary supplements and dietary patterns in managing gout is inconclusive, with only modest benefits observed in selected interventions. High-quality, well-designed randomised controlled trials are required to establish clear dietary recommendations for the management of gout.

## INTRODUCTION

Gout is an inflammatory arthritic condition characterised by elevated serum uric acid (SUA) concentrations, leading to the formation of monosodium urate (MSU) crystals.^1^ These crystals are deposited in joints, commonly the knee, fingers, and other peripheral sites, resulting in the clinical manifestations of gout.^2^ The phenotypic progression of gout begins with asymptomatic hyperuricemia, followed by acute gout flares, an intercritical phase, and ultimately chronic tophaceous gout.^3^ If left untreated or inadequately managed, it can result in impaired musculoskeletal function, occupational disability, and increased risk of comorbidities, including hypertension, cardiovascular disease, chronic kidney disease, and diminished health-related quality of life (HRQOL).^4^ Gout affects approximately 1–4% of the global population, with an incidence rate ranging from 0.1% to 0.3%.^5^ The prevalence of the condition is significantly higher in the male population, with a male-to-female ratio of three to ten.^5^

Purines are fundamental molecules required for the synthesis of nucleotides, which play a critical role in DNA and RNA formation as well as cellular energy transfer.^6^ While the majority of purines in the human body are of endogenous origin, a significant proportion is also obtained through dietary intake.^6^ Following metabolic processing, purines are ultimately broken down into uric acid, which is the final product of purine catabolism in humans.^7^ Under physiological conditions, uric acid is subject to rigorous regulation through a balance of production, reabsorption, and excretion.^8^ It has been determined that approximately 90% of filtered uric acid is reabsorbed, with the remaining portion being eliminated via the kidneys and, to a lesser extent, the intestines and gut microbiota.^7,8^ However, excessive dietary intake of purine-rich foods or impaired excretory function can disrupt this balance.^9,10^ Diet plays a pivotal role in modulating both uric acid synthesis and excretion, not only by influencing renal and intestinal elimination but also by affecting intestinal homeostasis and urate transport mechanisms.^11^

In the management of gout, dietary interventions have been shown to exert a complex influence on the manifestation of symptoms. A purine-rich diet,^12^ alcohol consumption,^13,14^ and sugar-sweetened beverages,^15^ have been shown to exacerbate gout symptomatology, whereas vegetarian, Dietary Approaches to Stop Hypertension (DASH), and Mediterranean diets have been reported to reduce the risk of hyperuricemia and gout.^16–20^ Cross-sectional studies have reported inverse associations between certain dietary components, such as cherry consumption,^21^ omega-3 fatty acid supplementation,^22^ and vitamin C intake.^23^ Accordingly, the European League Against Rheumatism (EULAR) has recommended dietary and lifestyle modifications as a means of reducing serum uric acid (SUA) concentrations and effectively managing acute attacks.^24^ However, the overall effectiveness of dietary patterns and supplements in the clinical management of gout remains uncertain.

The aim of the present systematic review was to synthesise all randomised controlled trials (RCTs) that evaluated the efficacy of dietary interventions or nutritional supplementation in patients diagnosed with gout.

## METHODS AND MATERIALS

### Systematic review protocol and PICO

**Figure 1.**
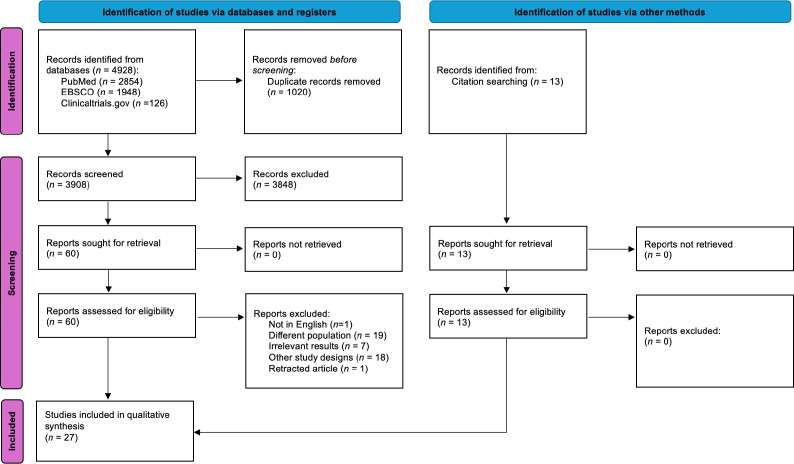
PRISMA.

This followed The Preferred Reporting Items for Systematic Reviews Analyses (PRISMA) (**Figure 1**).^25^ The study protocol was published on the Open Science Framework (OSF) website (https://osf.io/6c72e/). The research question in the PICO format is detailed in **Supplementary Table 1**.

### Search strategy and algorithm

Two independent reviewers (E.C.P. and A.G.) identified relevant studies through PubMed, EBSCO, and clinicaltrials.gov databases from the study’s inception until May 2025. Any disagreements between the reviewers were resolved by a senior reviewer (M.G.G.).

The Rayaan software,^26^ was utilised for the identification and elimination of duplicate records, in addition to the facilitation of the screening process. In the initial phase, titles and abstracts were screened to ascertain compliance with the established inclusion criteria. Records that passed this stage of the process were then subjected to a full assessment of their content to determine their final eligibility.

The search terms and keywords applied in the present study included “gout,” “gouty arthritis,” “diet,” “supplement,” and “dietary supplement.” The search was conducted in English only. The search syntax is presented in **Supplementary Table 2**.

### Inclusion and exclusion criteria

Studies were included in the synthesis if they (1) involved adult patients with gout, (2) were published until May 2025, (3) were written in English, (4) of any duration, (5) were RCTs, and (6) assessed any diet or dietary supplement alone or in comparison with other diets. Studies were excluded if they met any of the following criteria: (1) published in a language other than English; (2) focused on non-musculoskeletal and rheumatic diseases apart from gout; (3) animal or preclinical studies; (4) review articles, systematic reviews, or meta-analyses; (5) non-comparator studies (e.g., case reports); (6) non-randomised trials, cohorts, or cross-sectional studies; (7) did not evaluate any diet or dietary supplement as an intervention; and (7) included pediatric populations (children or adolescents).

### Outcomes of interest

The data extraction included study characteristics (first author’s surname, year of publication, journal and type of publication, clinical trial identifier, country of study, study design, funding source, and whether the study was multicentre) and details regarding the sample and method of randomisation. Additional extracted data included the intervention, comparator, study duration, analyses performed, dropouts, adverse events, and primary outcomes. All primary and secondary outcomes were considered as important, including SUA concentrations, frequency and symptomatology of gout flares, health-related quality of life, inflammation status, and dietary adherence.

### Quality assessment of the studies

The methodological quality of the included studies was assessed by two independent reviewers (A.G. and E.C.P.) using the Risk of Bias 2.0 (RoB) tool for RCTs,^27^ which evaluates potential bias in five domains: randomisation process, deviations from the intended interventions, missing outcome data, measurement of the outcome, and selection of the reported result. Any disagreements were resolved through discussions with a more experienced investigator (M.G.G.).

### Data synthesis

Owing to the substantial heterogeneity among the studies, a meta-analysis was deemed inappropriate. A systematic synthesis was performed to summarise the findings.

## RESULTS

### Search Results

Of the 4928 articles screened, 1020 were identified as duplicates and excluded from further consideration. The remaining 3908 articles were evaluated at the title and abstract levels. Sixty studies were subjected to full-text review. In addition, 13 studies were identified through citation searches. Twenty-seven studies satisfied the criteria for inclusion in the present systematic review.^28–53^ The lists of excluded studies are detailed in **Supplementary Table 3** and **Supplementary Table 4**.

### Studies characteristics

Of the studies included, 18 were found in full-text,^28–33,37,38,40,41,47–54^ two referred to posters based on the same work,^34,35^ and three were the clinical registries of the trials.^36,39,42^ Four studies referred solely to clinical trial registrations without published results at the time of this review.^43–46^ The diagnostic criteria for gout used in each study are detailed in **Supplementary Table 5** and **Supplementary Table 6**.

Three studies originated in New Zealand,^29,49,50^ eight in China,^30–33,48,52–54^ three in the United States of America (USA),^40–42,47^ one in Australia,^37^ one in the Netherlands,^34–36^ one in Denmark,^38,39^ and in one study, the country of origin was not reported.^28^ Ten of the studies were open-label,^33–36,38–42,47–50,53^ four were double-blinded,^29,31,32,51^ one was single-blinded,^37^ and for four studies, blinding was not reported.^28,30,52,54^

Nineteen studies assessed interventions with dietary supplements,^28–33,40,43–54^ (**Table 1** and **Table 2**) and four RCTs evaluated the effect of dietary patterns (**Table 3**).^34,35,37,38,41^ Among the studies with published results on dietary supplements, three evaluated cherry juice or extract,^28,40,47^ seven investigated traditional Chinese medicine (TCM) supplements alone,^30–33,52–54^ two assessed omega-3 supplementation,^50,51^ one examined skim milk powder (SMP) enriched with glycomacropeptide (GMP) and G600 milk fat extract,^29^ and one investigated vitamin C supplementation.^49^ One study randomised participants to receive either the external application of compound Qingbi granules (CQBG) (a TCM paste) diclofenac dimethylamine emulgel, or a control regimen consisting of a low-purine diet, increased water intake, oral loxoprofen, and sodium bicarbonate.^48^ Consistent with the interventions assessed, the comparator arms varied across studies. In cherry extract trials, comparators included pomegranate juice concentrate,^28^ or dietitian-assisted dietary modification for gout,^40,47^ TCM studies used comparators such as diclofenac dimethylamine,^54^ allopurinol,^33,52^ higher-dose allopurinol,^49^ pharmaceutical-grade olive oil,^51^ indomethacin,^53^ Tongfengding,^30^ a colchicine mimetic agent,^31^ placebo,^32^ or no intervention.^50^ Inclusion and exclusion criteria of each study included in the synthesis and concurrent gout treatment in studies are presented in **Supplementary Tables 7–10**.

**Table 1.** Characteristics of the included studies assessing TCM dietary supplements (part A).

**First author:**	Abhishek^51^	Dalbeth^29^	Schlesinger^28^	Singh^40,47^	Stamp^49^	Stamp^50^
**CTI**	ISRCTN79392964	ACTRN12609000479202	NR	N/A	ACTRN12610000545066	ACTRN12617000539336p
**Origin**	UK	New Zealand	USA - Israel	USA	New Zealand	New Zealand
**Publication**	*Rheumatol Adv Pract*, 2022 (full-text)	*Ann Rheum Dis.* 2012 (full-text)	*J Arthritis* 2012 (full-text)	(1) *J Clin Rheumatol*, 2020 (full-text) (2) *J Clin Rheumatol*, 2020 (full-text)	*Arthritis Rheum*, 2013 (full-text)	*BMC Rheumatol*, 2022 (full-text)
**Design**	Double-blind, parallel	Double-blind, parallel	Pilot	Open label, parallel	Open label, parallel	Open label, parallel
**Multicentre**	-	-	-	N/A	-	-
**Funding**	Versus Arthritis, Nottingham University Hospital NHS Trust	LactoPharma, the New Zealand Government Foundation for Research Science and Technologyv	Brownwood Acres Foods	UAB Center for Outcomes and Effectiveness Research and Education (COERE)/Minority Health Research Center, UAB Center for Clinical and Translational Studies	Health Research Council of New Zealand	Lottery Health New Zealand
**Randomisation**	Stratified randomisation, PC-generated, using permuted blocks of sizes 2 and 4 by a third party (1:1 ratio)	PC-generated sequence by an independent statistician (1:1:1 ratio)	Paper draw method	PC-generated permuted variable block design (1:1 ratio)	Stratified randomisation using blocks of size 4 (1:1 ratio)	Stratified randomisation, PC-generated, using permuted blocks of size 4 by an independent statistician (1:1 ratio)
**Patients**	N=60 with gout	N=120 with recurrent gout flares	N=18 with gout	N=84 with gout	N=40 with gout	N=40 with gout
**Intervention(s)**	Omega-3 fatty acid ethyl esters in each 1 g capsule (DHA: EPA 380mg:460mg) BID *(n*=30 )	SMP enriched with GMP and G600 (1.5 g GMP protein (10% total protein) and 0.525 g G600 (3.5% of total protein weight)) (*n*=40 )	Cherry juice concentrate BID (*n*=9 )	Cherry extract (1200 mg, 3 capsules/day) (*n*=41 )	Vitamin C (500 mg/day) (*n*=20 )	Omega 3 fish oil (6.2g/day) (*n*=19 )
**Comparator(s)**	Placebo: Capsules with pharmaceutical-grade olive oil (1g) BID (*n*=30 )	(1) Lactose powder control (*n*=40 ) (2) SMP control (*n*=40 )	Pomegranate juice concentrate BID (*n*=5 )	Dietitian-assisted diet modification for gout (*n*=43 )	Increase in the dose of allopurinol (*n*=20 )	No intervention (*n*=21 )
**Intervention duration**	28 weeks	3 months	4 months	9 months	8 weeks	6 months
**Primary outcomes**	Drop-out rate	Gout flare frequency	Gout flare frequency	Feasibility rates of the Internet study, patient satisfaction	SUA	SUA
**Secondary outcomes**	Recruitment rate, outcome data completeness, number of gout flares, severity and duration of gout flares between weeks 4 and 28, and study drug compliance, red blood cell, omega-3 fatty acid	TJC, SJC, SUA, fractional excretion of uric acid, CRP, patient global assessment of gout severity, HAQ-II.	Use of NSAIDs, SUA, serum creatinine	Gout flare frequency, HAQ, pain, dietary assessment, SUA, AEs	Plasma ascorbate and oxypurinol concentrations	Weight, BMI, HbA_1c_, cholesterol, HDL, and LDL concentrations, oxypurinol (for pts on allopurinol) mean number of gout flares, percentage of participants with flares, AEs
**Analysis**	ITT	ITT	PP	ITT	ITT	ITT
**Drop-outs**	*n*=1 in intervention, *n*=2 in the control group	*n*=2 (adverse events), *n*=8 (lost to follow up), *n*=8 (continued with no milk products due to adverse events)	*n*=2 in intervention, *n*=2 in the control group	*n*=2 in intervention, *n*=5 in the control group	None	*n*=5 in the intervention group (3 tolerance, 1 dabigatran initiation, 1 inconvenience)
**AEs**	Gastroenteritis, GI events, viral infection, ear, nose, and throat events, genitourinary, injury/accident, respiratory, nervous system, musculoskeletal in placebo group; GI, oral viral infection, eye, injury/accident, musculoskeletal in the intervention arm.	Diarrhea, nausea flatulence in all groups; gout flare, viral infection, prostate surgery in the SMP group; accidental fall, gout flare in the SMP/GMP/G600 group.	SUA, serum creatinine	Abdominal discomfort, nausea, vomiting, excessive burping, excessive passing gas, abdominal bloating, constipation, diarrhea, heartburn, loss of appetite, in both groups.	None	Cardiac disorders, GI disorders, general disorders, infections and infestations, injury, poisoning and procedural complications, investigations, musculoskeletal disorders, nervous system disorders, psychiatric disorders, respiratory disorders, skin and subcutaneous tissue disorders in intervention arm; cardiac disorders, GI disorders, general disorders, infections and infestations, injury, poisoning and procedural complications, musculoskeletal disorders, nervous system disorders, psychiatric disorders, respiratory disorders, skin and subcutaneous tissue disorders, ear and labyrinth, eye disorders, neoplasm, renal and urinary in control arm.
**Results**	The flare number, duration and time to 1st flare were comparable between groups. Compliance was high. Red blood cell and omega-3 fatty acid index increased twofold in the active arm and remained unchanged in the control arm.	Flare frequency decreased in all groups. SMP/GMP/G600 group had a greater reduction of gout flares, pain and increase in fractional excretion of uric acid. No difference in SJC, TJC, HAQ-II, patient global assessment, CRP, SUA between groups.	Cherry arm: flares per 4 mo reduced from 4.99 ± 1.53 to 1.56 ± 0.88 (p<0.05). 55% flare free, 5/9 discontinued NSAIDs. Pomegranate arm: flares reduced from 5.06 ± 2.83 to 3.60 ± 2.20, 20% flare free, none discontinued NSAIDs. SU and creatinine remained unchanged in both arms.	Internet-based interventions are feasible and patients were satisfied. Cherry extract showed more functional improvement than diet modification.	The reduction in SUA over 8 weeks was significantly less in vitamin C group compared to those who started or increased the dose of allopurinol.	Omega-3 supplementation did not show any effect on SUA concentrations, gout flares and BMI after 24 weeks. There was an inverse correlation between red cell omega-3 concentrations (total, EPA, DHA) and gout flares from week 12–24 with four participants in the omega-3 group reporting GI AEs likely related to supplementation.

AEs: adverse events; BID: twice daily; BMI: body mass index (kg/m2); CRP: C reactive protein; CTI: clinical trial identificatory; DHA: docosahexaenoic acid; EPA: eicosapentaenoic acid; ESR: erythrocyte sedimentation rate; GI: gastrointestinal; G600: G600 milk fat extract; GMP: glycomacropeptide; HAQ: health assessment questionnaire; ITT: intention-to-treat; kg: kilogram; m: meter; mo: months; N/A: not applicable; NR: not reported; NSAIDs: non-steroidal anti-inflammatory drugs; pts: patients; SMP: skim milk powder; SJC: Swollen joint count; SUA: serum urate; TJC: tender joint count.

**Table 2.** Characteristics of the included studies assessing TCM dietary supplements (part B).

**First author:**	Ren^48^	Renbin^52^	Shi^53^	Song^30^	Wang^31^	Wei^54^	Xie^32^	Yu^33^
**CTI**	ChiCTR1800018020	NR	NR	NR	ISRCTN65219941	NR	ChiCTR-TRC-12002245	ChiCTR-TRC-12001933
**Origin**	China	China	China	China	China	China	China	China
**Publication**	*Chin Med*, 2020; (full-text)	*J Tradit Chin Med*, 2008 (full-text)	*Chin J Integr Med*, 2008 (full-text)	*Chin J Integr Med*, 2008 (full-text)	*Int J Med Sci*, 2014 (full-text)	*Chin J Integr Med*, 2005 (full-text)	*Medicine (Baltimore),* 2017 (full-text)	*Trials,* 2018 (full-text)
**Design**	Open label, parallel, statistician blinded	NR	Open label, parallel	NR	Double-blind, double-dummy	NR	Double-blind, parallel	Open label, parallel, statistician blinded
**Multicentre**	-	-	-	-	-	-	Φ	Φ
**Funding**	National Natural Science Foundation of China, 2016 Open-end Fund of Education Ministry Key Laboratory for Research, Application of “Zang Xiang” Theory in Liaoning University of Traditional Chinese Medicine	NR	Bureau of Traditional Chinese Medicine of Jiangsu Province	Hubei Provincial Office of Science and Technology	Affiliated Hospital of Qingdao University Medical College	Project of Science and Technology Commission Foundation of Jiangsu Province in 1998	Zhejiang Province Natural Science Fund Committee, Ministry of Science and Technology of China	Physician Investigator Foundation of National TCM Clinical Research Base for Diabetes Mellitus
**Randomisation**	PC-generated numbers by an independent statistician (1:1 ratio)	“Randomly” NOD	PC-generated numbers and randomised grouping	By digital table	Block randomisation using a PC-generated random number tables (1:1)	Random number chart	Stratified block randomisation by study centre using PC-generated random numbers	PC-generated (1:1:1)
**Patients**	N=90 with acute gout	N=120 with gout	N=107 with acute gout	N=200 with gout	N=176 newly diagnosed acute gout	N=90 with acute gout and >=2 flares in the past year	N=210 males with intercritical or chronic gout	N=72 males with gout
**Intervention (s)**	CQBG topical paste (30 g of CQBG powder in 80 mL water) (*n*=29 ) (2) Diclofenac dimethylamine emulgel (*n*=28 )	Modified Simiao Tang (*n*=60 )	(1) Prescription I *(*n*=27 ) (2) Prescription II* (*n*=27 ) (3) Prescription III* (*n*=28 )	Weicao capsule 2 caps TID (*n*=100 )	Chuanhu (250 ml/day) + placebo (colchicine mimetic) (*n*=88 )	Rebixiao granule (2 packets TID) (*n*=60 )	Compound tufuling oral liquid 2 packs/day (*n*=139 )	(1) Yellow-dragon Wonderful-seed formula TID (*n*=24 ) (2) Yellow-dragon Wonderful-seed + gypsum decoction TID (*n*=24 )
**Comparator (s)**	Low-purine diet, increased water intake (>2000 mL/day), loxoprofen (60 mg), 1g sodium bicarbonate (*n*=27 )	Allopurinol (100 mg BID) (*n*=60 )	Indomethacin in 50 mg TID^ and benzobromarone 500mg QD (*n*=25 )	Tongfengding 2xTID (*n*=100 )	Colchicine 0.5 mg BID for 3 days and then QD + placebo (chuanhu mimetic) (*n*=88 )	Diclophenac sodium (25 mg/TID) (*n*=30 )	Placebo (*n*=71 )	Allopurinol 100 mg/day for one wk and then 200 mg/day (*n*=24 )
**Intervention duration**	7 days	1 month	2 weeks	4 weeks	10 days treatment, 12 weeks follow-up	RBXG 1 month after remission; Diclophenac 1 wk	12 weeks	4 weeks
**Primary outcomes**	VAS score in the target joint, onset time, pain improvement in the target joint, change in pain duration in the target joint, swelling score	Clinical effectiveness	Efficacy and safety of interventions	Red, swollen, hot and painful joints, condition of local motion	Recurrence rate of acute gouty arthritis	SUA	Average decrease and decrease rate of SUA	SUA
**Secondary outcomes**	SUA, CRP, synovium thickness (ultrasound)	SUA, CRP, blood and urine tests, LFT, KFT, safety evaluation	Heart rate, blood pressure, blood uric acid, routine blood, and routine urine, blood and lipid functions, LFT, KFT, arthritis	B_2_-microglobulin, 24-h urinary protein, hemoglobin, serum creatinine, creatinine clearance, BUN, SUA, total cholesterol, triglycerides	Changes in WBC and CRP	Arthralgia, tumefaction, tenderness, joint function, CRP, ESR	Decrease in the frequency of recurrent joint swelling or pain	Urine urate, SF-36, ESR, CRP, X-ray film
**Analysis**	PP	mITT	ITT	ITT	Both ITT and PP	ITT	Both ITT and PP	ITT and PP
**Drop-outs**	*n*=1 in CQBG group (pruritus), *n*=2 in diclophenac group (compliance), *n*=3 in the control group (compliance)	None	None	*n*=4 in intervention, *n*=8 in the control group	*n*=3 in intervention (increased stool frequency, blind broke, bitter taste), *n*=9 in the control group (diarrhea, liver damage, poor compliance)	None	*n*=25 in intervention, *n*=12 in the control group	*n*=7 in intervention, *n*=3 in the control group
**AEs**	Gastric or abdominal pain, edema, skin itch in intervention arm; gastric or abdominal pain in the control group	None	“Mobility attack” and stomachache in the control group.	Nausea, vomiting, poor appetite in both arms.	Diarrhea, nausea, vomiting in both groups	Nausea, vomiting, epigastrium distress, defecation with soft or loose stool in the intervention arm; nausea and vomiting in the control group.	Leucopenia in both groups	None
**Results**	CQBG significantly reduced VAS, pain duration, improvement onset, swelling and synovial thickness. No difference in CRP or SUA levels between groups.	The treatment group demonstrated a higher total effective rate compared to the control group (86.7% vs. 68.3%, p < 0.01). Reductions in SUA and CRP levels were greater in the treatment arm.	The clinical efficacy was higher in the intervention groups vs. the control group. SUA levels decreased in the Prescription II arm and in the control arm. Leucocytes decreased in the Prescription I arm. Arthritis decreased in all groups, the decrease was greater in intervention.	Weicao capsule improved renal function and decreased 24-h urinary protein, SUA, lipid and β2-microglobulin concentrations. SUA levels decreased in both arms.	Chuanhu was non-inferior to colchicine in lowering recurrence rates, CRP levels and WBC (recurrence rates 12.50% vs 14.77%; 95% CI: −10.78%, 6.23%).	RBXG lowered SUA levels. The total effective rate was 95% vs 90% in control arm.	CoTOL significantly reduced SUA concentrations and gout recurrence rates compared to control, with fewer AEs and no severe safety concerns.	Yellow-dragon Wonderful-seed did not reduce SUA levels. All groups had decreased the SUA concentrations. SF-36 and CRP levels did not differ between groups.

AEs: adverse events; BID: twice daily; BMI: body mass index (kg/m2); BUN: blood urea nitrogen; COBG: compound Qingbi granules; CoTOL: compound tufuling oral liquid; CRP: C-reactive protein; ITT: intention-to-treat; KFT: kidney function tests; kg: kilogram; LFT: liver function tests; m: meter; NOD: not-other defined; NR: not reported; QD: once daily; SF-36:36-Item Short Form Health Survey; SUA: serum uric acid; TCM: traditional Chinese medicine; TID: Three times per day; VAS: visual analog scale; WBC: white blood cells; wk: week(s).

*: Prescription I: Rhizoma Atractylodis 20 g, Cortex Phellodendri 20 g, Semen Coicis 30 g, Radix Cyathulae 30 g, Rhizoma Smilacis Glabrae 30 g, Caulis Lonicerae 20 g, and Radix Paeoniae rubra 20 g; Prescription II: Rhizoma Atractylodis 20 g, Cortex Phellodendri 20 g, Semen Coicis 30 g, Radix Cyathulae 30 g, Gypsum Fibrosum 50 g, Rhizoma Anemarrhenae 20 g, and Ramulus Cinnamomi 5 g; Prescription III: Rhizoma Atractylodis 20 g, Cortex Phellodendri 20 g, Semen Coicis 30 g, Radix Cyathulae 30 g, Rhizoma Smilacis Glabrae 30 g, Caulis Lonicerae 20 g, Radix Paeoniae rubra 20 g, Gypsum Fibrosum 50 g, Rhizoma Anemarrhenae 20 g, and Ramulus Cinnamomi 5 g.

^: When the symptoms improved, the dose was reduced to 25 mg, 3 to 4 times a day.

**Table 3.** Characteristics of the included studies assessing dietary plans.

**First author:**	Christensen^38,39^	Holland^37^	Juraschek^41,42^	Kretova^34–36^
**CTI**	NCT03664167	NR	NCT03569020	NL74142.029.20
**Origin**	Denmark	Australia	USA	The Netherlands
**Publication**	*Arthritis Rheumatol,* 2024; (full-text)	*Intern Med J*, 2015; (full-text)	*Nutrients,* 2021; (full-text)	(1) *Am Med J Rheumatol,* 2024 (poster) (2) *Arthritis Rheumatol,* 2024 (poster)
**Design**	Open-label, parallel	Single blind, parallel	Open-label, cross-over	Open-label, parallel
**Multicentre**	-	Φ	-	-
**Funding**	Sheffield Hallam University	None	Rheumatology Research Foundation, Johns Hopkins Institute for Clinical and Translational Research, NIH-funded	Vermeer 14 Foundation
**Randomisation**	PC-generated randomization using permuted block sizes of 2 to 4. Randomization was stratified by sex (male vs. female), baseline morbid obesity (BMI < 40 vs. ≥ 40 kg/m²), and baseline serum urate (SU < 6 vs. ≥ 6 mg/dL) (1:1 ratio)	PC-generated using block design in the number of 10 (1:1 ratio)	PC-generated block randomisation with variable block sizes (2 and 4), prepared by a biostatistician (1:1 ratio)	NR
**Patients**	N= 61 patients with gout	N= 30 patients with gout	N= 43 patients with gout	Patients with gout (*n*= 31 )
**Intervention(s)**	Initial Phase (Weeks 1–8): Dietitian-supervised hypocaloric full meal–replacement diet providing 800–1,000 kcal/day using products from the Cambridge Weight Plan. Tapering Phase (Weeks 9–16): Transition to a fixed-energy diet providing 1,200 kcal/day, incorporating up to two meal replacement products daily (*n*=29 )	Dietary advice, in line with the British Society for Rheumatology guidelines for the management of gout (*n*=14 )	DDG: $105 of groceries patterned after the DASH diet (*n*=21 )	Mediterranean-style whole food plant-based diet (*n*=18 )
**Comparator(s)**	Three group-based sessions with a dietician in which participants received basic nutritional advice according to the Danish National Health Authority (*n*=32 )	Advice regarding the importance of compliance with drug therapy, the benefit of weight loss, exercise and reduced alcohol intake (*n*=15 )	Self-directed grocery shopping (*n*=22 )	Usual care (*n*=15 )
**Intervention duration**	16 weeks	6 months	4 weeks	16 weeks
**Primary outcomes**	Change in body weight	Change in SUA levels	Change in SUA levels	Change in SUA levels
**Secondary outcomes**	SUA levels, fatigue, pain, HAQ, SF-36 Mental Component Summary and Physical Component summary, patient global assessment, number of gout flares, SJC, TJC, change in number of tophi from baseline	Change in number of flares, dietary modification or attempted weight loss and knowledge improvement	Number of gout flares, pain, BMI, TUG, HDL, LDL, TC, triglycerides, glucose, GFR, adverse events	Disease activity, cardiovascular risk factors (HDL, LDL, SBP, DBP, HbA1_c_, TC, fasting blood glucose ), BMI, WC
**Analysis**	ITT	NR	ITT	ITT
**Drop-outs**	*n*=4 in the intervention, *n*=9 in the control arm	*n*=2 in the intervention group	None	*n*=1 in the intervention, *n*=3 in the control arm
**AEs**	Tiredness, flatulence, and back pain among others	NR	Hunger, bloating, diarrhea, thirst, fatigue, headache, lightheadedness, nausea in the control group	No serious AEs
**Results**	The intensive diet decreased their body weight. There were no differences in SUA, SF-36, HAQ, VAS patient global assessment, pain and fatigue after the intervention.	SUA did not change after the intervention. The intervention group improved its knowledge and dietary behavior.	In the first period, DDG reduced SUA; however, in the second period this effect was lost. DDG reduced urine sodium excretion, was easy to follow and was well tolerated.	The Mediterranean-style diet decreased SUA, gout severity and pain (as shown by VAS), BMI, WC, LDL. Gout flares were similar among the two groups.

AEs: adverse events; BMI: body mass index; DDG: Dietitian-directed groceries; GFR: Glomerular filtration rate; HAQ: health assessment questionnaire; HbA_1c_: hemoglobin A1_C_; HDL: high-density lipoprotein; NR: not reported; LDL: low-density lipoprotein; SBP: systolic blood pressure; DBP: diastolic blood pressure; SF-36:36-Item Short Form Health Survey; SJC: swollen joint count; SUA: serum uric acid; TC: total cholesterol; TJC: tender joint count; TUG: timed up and go; WC: waist circumference.

Among the studies evaluating dietary patterns, one implemented a Mediterranean-style whole food plant-based diet,^34–36^ one provided dietary advice aligned with the British Society for Rheumatology guidelines for the management of gout,^37^ one implemented hypocaloric diet plans,^38,39^ and one used dietitian-directed groceries based on the DASH diet.^41,42^ The comparator arms were usual care,^34–36^ more generic dietary and lifestyle advice,^37–39^ and self-directed grocery shopping,^41,42^ respectively.

### Outcomes of Interest

The majority of studies assessed changes in SUA concentrations,^29–42,47–50,52–54^ frequency of gout flares,^28,29,31,32,37–42,47,50,51^ and joint symptoms such as the number of red, tender, or swollen joints.^29,30,32,33,53,54^ Pain was commonly assessed using various scales, including a 0–10 Likert scale,^40,47^ visual analogue scales (VAS),^38,39,48^ or pain-related subscales from the Western Ontario and McMaster Universities Osteoarthritis Index (WOMAC).^41,42^

Shi et al.^53^ and Singh et al.^40,47^ examined the efficacy and safety of TCM interventions, feasibility of web-based study designs, and patient satisfaction. Abhishek et al.^51^ specifically assessed participant drop-out rates. Markers of systemic inflammation were reported in several studies, including C-reactive protein (CRP) concentrations,^29,31,33,48,52,54^ erythrocyte sedimentation rate (ESR),^33,54^ and white blood cell (WBC) count.^31^

Indicators of cardiometabolic health and glucose regulation were commonly evaluated and included total cholesterol concentrations,^30,31,34–36,41,42^ high-density lipoprotein (HDL), and low-density lipoprotein (LDL) cholesterol levels,^34–36,41,42,50^ triglycerides concentrations,^30,41,42^ haemoglobin A1c (HbA1c),^34–36,50^ and arterial blood pressure.^34–36,53^

Anthropometric measurements primarily involved assessments of body mass index (BMI),^34–36,41,42,50^ and waist circumference.^34–36^

Quality of life of patients was assessed using the health assessment questionnaire (HAQ),^29,38–40,47^ as well as the 36-Item Short Form Health Survey (SF-36).^38,39^

While the majority of the studies assessing dietary supplements evaluated gout flare frequency^28,29^ and SUA levels as primary outcomes,^49,50^ others focused on feasibility,^40,47^ and drop-out rates.^51^ Most studies assessing TCM supplementation had as primary outcomes gout-related symptoms including pain,^48^ swollen joints,^30,48^ and recurrence,^31^ SUA concentrations^32,33,54^, and the efficacy and effectiveness of proposed interventions.^52,53^ The trials assessing dietary patterns focused on SUA levels,^34–37,41,42^ while one investigated change (Δ) in body weight, as the primary outcome.^38,39^

### Adverse events

Several studies have reported adverse events, particularly gastrointestinal (GI) symptoms, such as diarrhea and nausea. These effects were noted in interventions including SMP/GMP/G600 (containing 1.5 g of GMP protein and 0.525 g of G600),^29^ cherry extract (3,600 mg/day),^40,47^ Rebixiao granules (RBXG; two packets, three times daily),^54^ Weicao capsules (1.5 g),30 and Chuanhu (250 mL/day),^31^ as well as in the control arms of these studies.

Additional reports of GI and abdominal discomfort were associated with interventions involving cherry extract,^40,47^ a topical cream containing compound CQBG (30 g of CQBG powder in 80 mL water),^48^ and omega-3 fatty acid supplementation—both at a high dose (6.2 g/day),^50^ and a moderate dose (1 g/day, with docosahexaenoic acid:eicosapentaenoic acid [DHA:EPA] at 380 mg:460 mg).^51^ Importantly, these adverse events were not exclusive to the intervention arms, as comparable GI symptoms were also observed in the respective control groups.

Other adverse events included vomiting, which was reported following the administration of cherry extract,^40,47^ Weicao capsules,^30^ Chuanhu,^31^ and RBXG,^54^ as well as poor appetite, which was noted during both the Weicao^30^ supplementation and cherry extract groups,^40,47^ including some control participants. Edema and skin itching were reported following the use of the CQBG topical paste,^48^ while musculoskeletal complaints were observed in both studies involving n-3 fatty acid supplementation.^50,51^ One study32 reported leucopenia in both study arms.

Christensen et al. observed tiredness, flatulence, and back pain, among other symptoms, in all comparator arms, including both hypocaloric diets in the intervention arm, and dietetic advice in the control group.^38,39^ Furthermore, Juraschek et al. noted hunger, bloating, diarrhea, thirst, fatigue, headache, lightheadedness, and nausea, but only among controls.^41,42^ The studies assessing dietary interventions did not report any other adverse events.

### Efficacy of interventions

**Figure 2.**
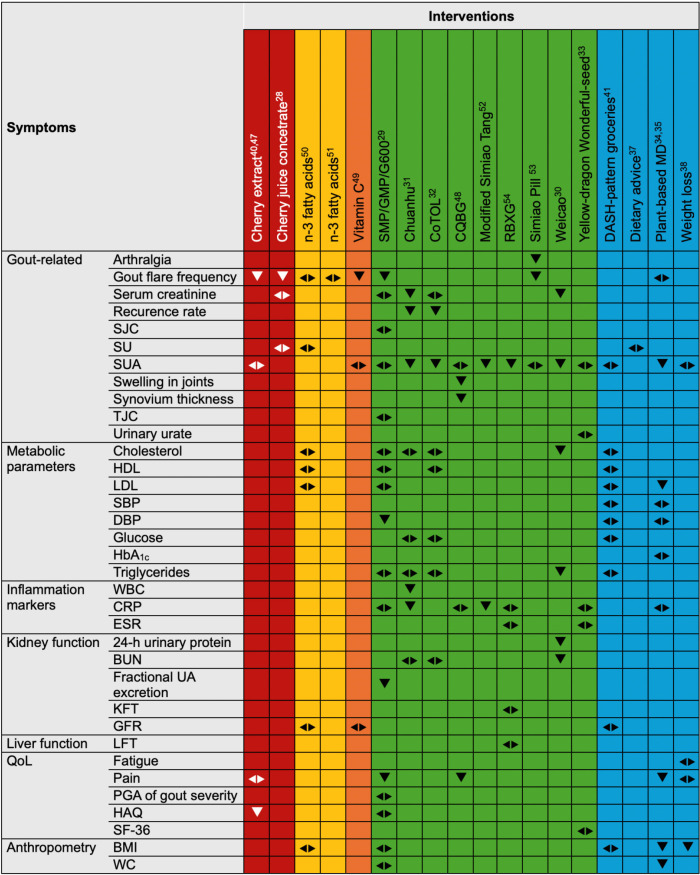
Effect of dietary intervention and dietary supplements on gout-specific outcomes.^*,†^ AEs: adverse events; BMI: body mass index (kg/m^2^); BUN: blood urea nitrogen; CoTOL: compound tufuling oral liquid; CRP: C-reactive protein; DASH: Dietary Approaches to Stop Hypertension; DBP: diastolic blood pressure; ESR: erythrocyte sedimentation rate; GFR: glomerular filtration rate; GMP: glycomacropeptide; HAQ: health assessment questionnaire; HbA_1c_: hemoglobin A_1C_; HDL: high-density lipoprotein; KFT: kidney function tests; kg: kilogram; LDL: low-density lipoprotein; LFT: liver function tests; m: meter; MD: Mediterranean diet; PGA: patient global assessment; RBXG: Rebixiao granule; SBP: systolic blood pressure; SF-36: 36-Item Short Form Health Survey; SJC: swollen joint count; SMP: skim milk powder; SU: serum urate; SUA: serum uric acid; TJC: tender joint count; UA: uric acid; WBC: white blood cells; WC: waist circumference; * Colors denote different interventions (red for cherry-related interventions, yellow for n-3 fatty acids, orange for vitamin C, green for Traditional Chinese Medicine and blue for dietary patterns); † ◄►no change; □ decreased.

As detailed in **Figure 2**, interventions with Chuanhu,^31^ modified Simiao Tang,^52^ RBXG,^54^ Weicao capsules,^30^ and the plant-based Mediterranean style diet,^34,35^ were associated with reductions in SUA concentrations. Gout flares were improved when interventions with cherry were implemented (extract^40,47^ or juice concentrate28), SMP/GMP/G600,^29^ and Simiao Pill (Prescription III).53 Recurrence rates were less frequent following treatment with Chuanhu^31^ or CoTOL32 TCM supplements. Regarding joint swelling and synovial thickness, TCM CQBG supplements were proved effective in reducing these symptoms.^48^

Most metabolic parameters remained stable despite the interventions; however, the Mediterranean-style plant-based diet lowered LDL concentrations,^34,35^ Weicao reduced total cholesterol and triglycerides levels,^30^ and SMP/GMP/G600 decreased diastolic blood pressure.^29^ BMI was improved through both weight loss interventions,^38^ as well as with the plant-based Mediterranean diet, with the latter also leading to a decrease in waist circumference of patients.^40,47^

CRP concentrations were improved through the administration of both Chuanhu,^31^ and modified Simiao Tang^52^ TCM formulas. In terms of renal health, the Weicao capsule led to decreases in 24-hour urinary protein and blood urea nitrogen levels.^30^

Regarding the quality of life of patients with gout, reductions in pain were observed in various interventions, including supplementation with SMP/GMP/G600,^29^ and CQBG^48^, as well as with the Mediterranean-style plant-based diet.^34,35^ Supplementation with cherry extract also induced improvements in HAQ scores.^40,47^

### Risk of bias

**Figure 3.**
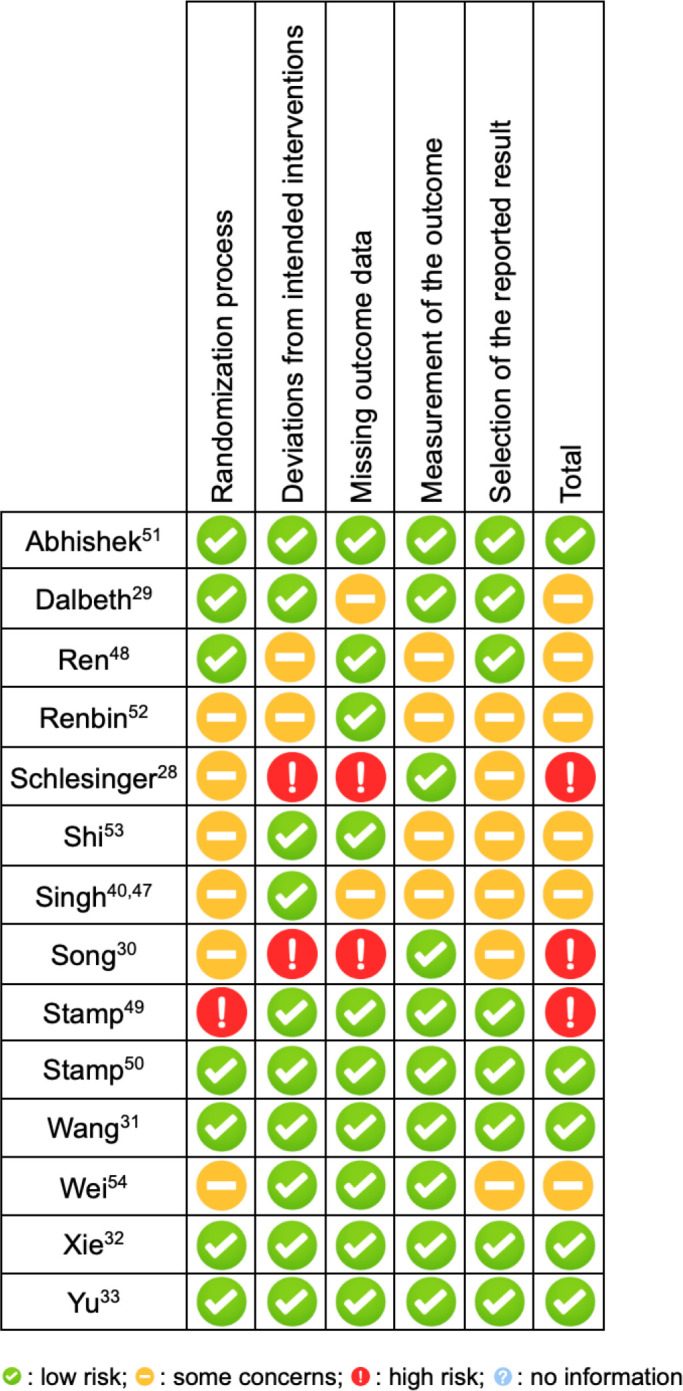
Risk of bias of the studies included in the synthesis assessing dietary supplements.

**Figure 4.**
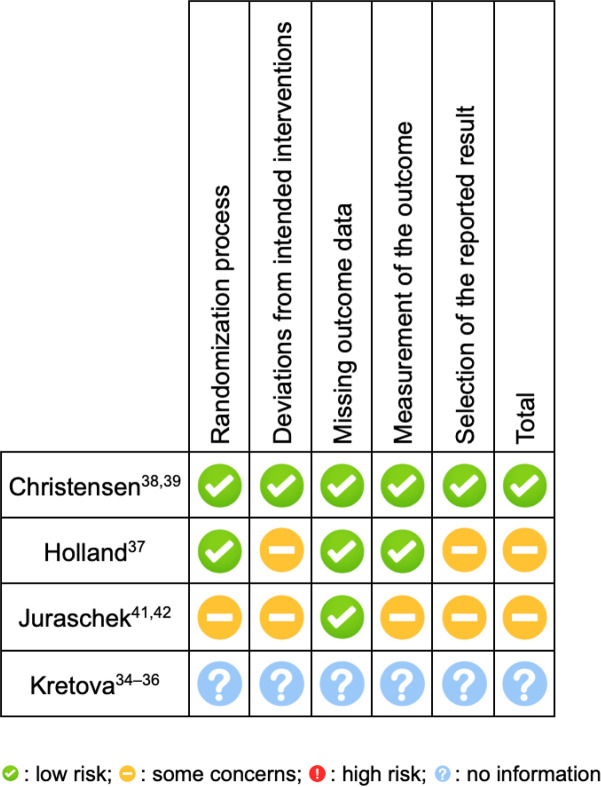
Risk of bias of the studies included in the synthesis assessing dietary patterns.

A summary of the quality assessment of the included RCTs on dietary supplements and dietary patterns is shown in **Figure 3** and **Figure 4**, respectively. Among the RCTs examining dietary supplements, approximately half of the assessed domains were rated as presenting some concerns,^29,40,47,48,52–54^ while three were classified as high risk,^28,30,49^ and five as low risk of bias.^31–33,50,51^ In the case of dietary pattern interventions, one study was rated as having low risk of bias,^38,39^ two exhibited some concerns,^37,41,42^ and one could not be fully appraised due to insufficient information, as the assessment was based solely on a registry protocol and abstract.^34–36^

### Ongoing Research

**Table 4** presents ongoing or unpublished RCTs registered in clinicaltrials.gov that investigated the use of dietary supplements in the management of gout. Two studies assess the effects of tart cherry, one evaluating tart cherry juice,^45,46^ and the other examining tart cherry extract.^43^ Another study investigates the role of probiotics in gout management.^44^ These trials originate from three different countries, namely the United Kingdom (UK),^45,46^ the USA,^43^ and China.^44^ Key endpoints include changes in gout flare frequency and intensity,^44–46^ SUA,^44–46^ and CRP^43^ concentrations.

**Table 4.** RCTs without any published results to date.

**Study name/first author**	**Study Origin**	**Allocation**	**Intervention(s)**	**Reported Outcomes**	**Randomised (N)**	**Funding**
Lamb^45,46^	UK	NR	Tart cherry juice	Change in gout flare frequency, intensity, SUA levels, fractional excretion of uric acid, inflammatory markers, oxidative damage, antioxidant status, blood pressure, arterial stiffness, lipid profile, BMI	120	National Research Ethics Service, Yorkshire and The Humber-Leeds West Research Ethics Committee
Schlesinger^43^	USA	1:1:1	(i) Tart cherry extract 60 mL (ii) Tart cherry extract 120 mL	CRP levels, AUC for plasma anthocyanin concentration, Nrf2 expression, peak plasma anthocyanin concentration, plasma anthocyanin half-life	10	Rutgers, The State University of New Jersey
The Affiliated Hospital of Inner Mongolia Medical University^44^	China	1:2	Probiotics	SUA levels, acute attack of gout	300	The Affiliated Hospital of Inner Mongolia Medical University

AUC: area under the curve; BMI: body mass index; CRP: C-reactive protein; Nrf2: Nuclear factor erythroid 2-related factor 2; RCTs: randomised controlled trials; SUA: serum uric acid.

## DISCUSSION

A careful evaluation of the RCTs included in the synthesis revealed considerable heterogeneity among the studies, particularly in terms of study design and reported outcomes. Both dietary supplementation and diet-based interventions yielded different results with respect to gout recurrence and symptomatology, flare frequency, SUA, anthropometry, quality of life, and biomarkers, including CRP and ESR.

### Efficacy of Dietary Supplementation

Regarding gout manifestations and disease severity, dietary supplementation yielded varying outcomes. SUA concentrations presented greater reduction after modified Simiao Tang,^52^ RBXG,^54^ compound tufuling oral liquid (CoTOL),^32^ and in an intervention arm of the study of Shi et al.^53^ Additionally, Dalbeth et al*.*^29^ reported increased fractional excretion of uric acid, which might be related to decreased SUA concentrations,^55^ although this association was not explicitly established in their study. Nevertheless, apart from these instances, most interventions did not demonstrate superior efficacy over control conditions in lowering SUA. Importantly, it should be noted that both elevated,^56^ and normal SUA concentrations,^57^ have been observed during gout flare episodes, suggesting that factors beyond uric acid concentration alone may play a significant role in triggering flares.

TCM approaches that were examined in this review employed therapeutic strategies to address dampness-heat syndrome,^30,33,48,52–54^ primarily by regulating the Pi (Spleen) function,^54^ and leveraging its anti-inflammatory effects to reduce joint pain and swelling.^31,32^ According to TCM theory, the pathogenic mechanism underlying these conditions involves internal dampness-heat, which contributes to blood stagnation.^58^ This internal imbalance fosters the formation of phlegm and stasis, ultimately impairing several of the primary Zang (solid) organs.^58^ Importantly, these organ systems are not understood anatomically, but as interconnected functional entities that integrate physiological, emotional, and energetic domains.^59^

Clinically, while some TCM formulations demonstrated improvements in specific outcomes, such as reduced joint swelling,^48^ synovial thickness,^48^ and arthritis,^53^ lower recurrence rates,^32^ and improved renal function with Weicao capsule.^30^ Overall anti-inflammatory effects were modest,^31,33,48^ with the exception of modified Simiao Tang.^52^ Notably, Yellow-dragon Wonderful-seed did not significantly reduce urinary urate concentrations.^33^ Reduced kidney function is a well-established risk factor for hyperuricemia due to impaired urate clearance,^60,61^ while increased SUA concentrations may facilitate MSU crystal deposition in extra-articular sites such as the blood vessels and kidneys contributing to both localised and systemic inflammation.^61^ The inflammatory cascade, driven by activated macrophages and neutrophils, results in the release of pro-inflammatory cytokines during acute gout flares.^62–64^ Although elevated CRP concentrations have been associated with flare recurrence, the exact nature of this relationship remains unclear.^65^ Nevertheless, CRP is recognised as a sensitive and specific biomarker for assessing disease activity in gout.^66^ Among the studies included, a significant reduction in CRP was observed only following the use of modified Simiao Tang,^52^ while other interventions reported no alterations.^29,31,33,48^ Pain reduction was reported along with a decrease in gout flares and recurrence,^29^ and improvement in onset, swelling, and synovial thickness.^48^ These effects were accompanied by decreases in inflammatory markers.^29,48^ Flare frequency was reduced after SMP/GMP/G600,^29^ and cherry extract,^28^ while CoTOL supplementation contributed to lower recurrence rates.^32^

Despite some symptomatic and physiological improvements, the impact of TCM on the broader HRQOL remains uncertain. Yu et al*.* reported no significant improvements in HRQOL following TCM supplementation.^33^ Similarly, no changes were observed in standardised outcome measures such as the HAQ-II following SMP/GMP/G600 intervention,^29^ and SF-36 following Yellow-dragon Wonderful-seed.^33^ These findings are particularly relevant, given that physical functioning in individuals with gout is frequently impaired, especially among those who regard gout as their primary health concern.^67^ Furthermore, HRQOL has been consistently associated with disease-specific factors such as flare frequency, polyarticular involvement, pain severity, and the presence of widespread pain.^68^

### Role of Diet-Based Interventions

Dietary interventions, particularly those modeled on the DASH and Mediterranean-style diets, have shown promise in reducing SUA concentrations,^34,35,41^ as well as alleviating gout severity and associated pain.^34,35^ Both dietary patterns emphasise the consumption of anti-inflammatory components, including a high intake of fruits, vegetables, and low-fat dairy products, while reducing saturated and total fat intake.^17,19^ These dietary frameworks have been associated with a lower incidence of hyperuricemia,^17,69^ suggesting their potential role in both prevention and management of gout. A plant-based Mediterranean diet decreased cardio-metabolic parameters such as systolic blood pressure, LDL-cholesterol and HbA_1c_.^34,35^

However, not all dietary interventions yield favorable outcomes. Interventions that relied solely on dietary advice showed limited efficacy, with approximately half of the participants unable to implement or sustain meaningful dietary changes.^37^ Weight loss efforts have also been inconsistent. While general weight reduction did not lead to significant improvements in gout symptomatology,^38^ some evidence suggests that intensive low-energy diet regimens may produce short-term reductions in SUA concentrations. Nevertheless, the long-term sustainability of such approaches remains uncertain, as their effectiveness in maintaining lowered SUA concentrations over time appears limited.^38^

### Limitations of the study

This review has several limitations. First, the scope of the literature search was restricted to English-language RCTs, which may have led to the exclusion of relevant studies published in other languages. Given the prominence of TCM in gout management in Chinese clinical settings, Chinese-language publications could represent a valuable body of evidence that warrants further systematic exploration. However, due to our limited knowledge of the Chinese language, we were unable to evaluate studies published in Chinese with confidence in their accuracy and integrity.

Secondly, the methodological quality of the included RCTs was generally low, which poses challenges for conducting robust comparative analyses and limits the strength of the conclusions drawn. Many of the studies exhibited risks of bias due to issues such as inadequate randomisation procedures, incomplete outcome data, and selective reporting. These methodological flaws hinder the ability to perform rigorous comparative analyses, increase the potential for confounding variables, and reduce the reliability of the findings. Furthermore, due to the highly heterogeneous nature of the studies included in the synthesis, it was not possible to perform a meta-analysis or apply a formal GRADE assessment. While GRADE would certainly add valuable insight, the wide variation in intervention types, alongside the differing comparator arms (e.g., placebo vs. allopurinol, colchicine, etc.), might influence the observed effects and lead to misleading conclusions.

## CONCLUSION

The present systematic review revealed inconsistent findings regarding dietary supplements and dietary patterns. While certain supplements and specific dietary models have demonstrated potential for improving disease outcomes, others have not shown significant effects on clinical manifestations. Given the rising prevalence,^70^ and economic burden of gout,^71^ exploring alternative approaches beyond medication has become increasingly important.^19,72^ More high-quality studies are required to rigorously evaluate these interventions in the context of gout management, address this inflammatory form of arthritis and alleviate the associated pain and functional impairments experienced by affected individuals.

## References

[1] Taylor WJ Fransen J Jansen TL Dalbeth N Schumacher HR Brown M Study for Updated Gout Classification Criteria: Identification of Features to Classify Gout. Arthritis Care Res (Hoboken) 2015;67:1304–15. 10.1002/acr.22585. 25777045 PMC4573373

[2] Dalbeth N House ME Aati O Tan P Franklin C Horne A Urate crystal deposition in asymptomatic hyperuricaemia and symptomatic gout: a dual energy CT study. Ann Rheum Dis 2015;74:908–11. 10.1136/annrheumdis-2014-206397. 25637002

[3] Bardin T Richette P. Definition of hyperuricemia and gouty conditions. Curr Opin Rheumatol 2014;26:186–91. 10.1097/BOR.0000000000000028. 24419750

[4] Khanna PP Nuki G Bardin T Tausche A-K Forsythe A Goren A Tophi and frequent gout flares are associated with impairments to quality of life, productivity, and increased healthcare resource use: Results from a cross-sectional survey. Health Qual Life Outcomes 2012;10:117. 10.1186/1477-7525-10-117. 22999027 PMC3499162

[5] Singh JA Gaffo A. Gout epidemiology and comorbidities. Semin Arthritis Rheum 2020;50:S11–6. 10.1016/j.semarthrit.2020.04.008. 32620196

[6] Carrey EA Perrett D Simmonds HA. Nucleic Acids, Purine, and Pyrimidine Nucleotides and Nucleosides: Physiology, Toxicology, and Dietary Sources. Encycl. Hum. Nutr., vol. 3–4, Elsevier; 2013, p. 189–96. 10.1016/B978-0-12-375083-9.00191-4.

[7] Maiuolo J Oppedisano F Gratteri S Muscoli C Mollace V. Regulation of uric acid metabolism and excretion. Int J Cardiol 2016;213:8–14. 10.1016/j.ijcard.2015.08.109. 26316329

[8] Sorensen LB Levinson DJ. Origin and extrarenal elimination of uric acid in man. Nephron 1975;14:7–20. 10.1159/000180432. 1124137

[9] Aihemaitijiang S Zhang Y Zhang L Yang J Ye C Halimulati M The Association between Purine-Rich Food Intake and Hyperuricemia: A Cross-Sectional Study in Chinese Adult Residents. Nutrients 2020;12:3835. 10.3390/nu12123835. 33334038 PMC7765492

[10] Cicero AFG Fogacci F Di Micoli V Angeloni C Giovannini M Borghi C. Purine Metabolism Dysfunctions: Experimental Methods of Detection and Diagnostic Potential. Int J Mol Sci 2023;24:7027. 10.3390/ijms24087027. 37108190 PMC10138451

[11] Yin H Liu N Chen J. The Role of the Intestine in the Development of Hyperuricemia. Front Immunol 2022;13:845684. 10.3389/fimmu.2022.845684. 35281005 PMC8907525

[12] Zhang Y Chen C Choi H Chaisson C Hunter D Niu J Purine-rich foods intake and recurrent gout attacks. Ann Rheum Dis 2012;71:1448–53. 10.1136/annrheumdis-2011-201215. 22648933 PMC3889483

[13] Zhang Y Woods R Chaisson CE Neogi T Niu J McAlindon TE Alcohol consumption as a trigger of recurrent gout attacks. Am J Med 2006;119:800.e13–8. 10.1016/j.amjmed.2006.01.020. 16945617

[14] Neogi T Chen C Niu J Chaisson C Hunter DJ Zhang Y. Alcohol quantity and type on risk of recurrent gout attacks: an internet-based case-crossover study. Am J Med 2014;127:311–8. 10.1016/j.amjmed.2013.12.019. 24440541 PMC3991555

[15] Choi HK Curhan G. Soft drinks, fructose consumption, and the risk of gout in men: prospective cohort study. BMJ 2008;336:309–12. 10.1136/bmj.39449.819271.BE. 18244959 PMC2234536

[16] Chiu THT Liu C-H Chang C-C Lin M-N Lin C-L. Vegetarian diet and risk of gout in two separate prospective cohort studies. Clin Nutr 2020;39:837–44. 10.1016/j.clnu.2019.03.016. 30955983

[17] Rai SK Fung TT Lu N Keller SF Curhan GC Choi HK. The Dietary Approaches to Stop Hypertension (DASH) diet, Western diet, and risk of gout in men: prospective cohort study. BMJ 2017;357:j1794. 10.1136/bmj.j1794. 28487277 PMC5423545

[18] Kontogianni M Chrysohoou C Panagiotakos D Tsetsekou E Zeimbekis A Pitsavos C Adherence to the Mediterranean diet and serum uric acid: the ATTICA study. Scand J Rheumatol 2012;41:442–9. 10.3109/03009742.2012.679964. 22827465

[19] Stamostergiou J Theodoridis X Ganochoriti V Bogdanos D Sakkas L. The role of the Mediterranean diet in hyperuricemia and gout. Mediterr J Rheumatol 2018;29:21–5. 32185293 10.31138/mjr.29.1.21 PMC7045958

[20] Guasch-Ferre M Bullo M Babio N Martinez-Gonzalez MA Estruch R Covas M-I Mediterranean Diet and Risk of Hyperuricemia in Elderly Participants at High Cardiovascular Risk. J Gerontol A Biol Sci Med Sci 2013;68:1263–70. 23599357 10.1093/gerona/glt028

[21] Zhang Y Neogi T Chen C Chaisson C Hunter DJ Choi HK. Cherry consumption and decreased risk of recurrent gout attacks. Arthritis Rheum 2012;64:4004–11. 10.1002/art.34677. 23023818 PMC3510330

[22] Zhang M Zhang Y Terkeltaub R Chen C Neogi T. Effect of Dietary and Supplemental Omega-3 Polyunsaturated Fatty Acids on Risk of Recurrent Gout Flares. Arthritis Rheumatol (Hoboken, NJ) 2019;71:1580–6. 10.1002/art.40896. PMC6717014 30908893

[23] Zou Y Liu Y Li S. Association between dietary vitamin C intake and gout among American adults. Front Immunol 2024;15:1431323. 10.3389/fimmu.2024.1431323. 39346908 PMC11437527

[24] Richette P Doherty M Pascual E Barskova V Becce F Castañeda-Sanabria J 2016 updated EULAR evidence-based recommendations for the management of gout. Ann Rheum Dis 2017;76:29–42. 10.1136/annrheumdis-2016-209707. 27457514

[25] Page MJ McKenzie JE Bossuyt PM Boutron I Hoffmann TC Mulrow CD The PRISMA 2020 statement: an updated guideline for reporting systematic reviews. BMJ 2021:n71. 10.1136/bmj.n71. 33782057 PMC8005924

[26] Ouzzani M Hammady H Fedorowicz Z Elmagarmid A. Rayyan—a web and mobile app for systematic reviews. Syst Rev 2016;5:210. 10.1186/s13643-016-0384-4. 27919275 PMC5139140

[27] Sterne JAC Savović J Page MJ Elbers RG Blencowe NS Boutron I RoB 2: a revised tool for assessing risk of bias in randomised trials. BMJ 2019:l4898. 10.1136/bmj.l4898. 31462531

[28] Schlesinger N. Pilot Studies of Cherry Juice Concentrate for Gout Flare Prophylaxis. J Arthritis 2012;01. 10.4172/2167-7921.1000101. 23334899

[29] Dalbeth N Ames R Gamble GD Horne A Wong S Kuhn-Sherlock B Effects of skim milk powder enriched with glycomacropeptide and G600 milk fat extract on frequency of gout flares: a proof-of-concept randomised controlled trial. Ann Rheum Dis 2012;71:929–34. 10.1136/annrheumdis-2011-200156. 22275296

[30] Song E Xiang Q Ren K Hu J Wu F Gong M Clinical effect and action mechanism of Weicao Capsule (威草胶囊) in treating gout. Chin J Integr Med 2008;14:103–6. 10.1007/s11655-008-0103-7. 18679600

[31] Wang Y Wang L Li E Li Y Wang Z Sun X Chuanhu Anti-Gout Mixture versus Colchicine for Acute Gouty Arthritis: A Randomized, Double-Blind, Double-Dummy, Non-Inferiority Trial. Int J Med Sci 2014;11:880–5. 10.7150/ijms.9165. 25013367 PMC4081309

[32] Xie Z Wu H Jing X Li X Li Y Han Y Hypouricemic and arthritis relapse-reducing effects of compound tufuling oral-liquid in intercritical and chronic gout. Medicine (Baltimore) 2017;96:e6315. 10.1097/MD.0000000000006315. 28296744 PMC5369899

[33] Yu XN Wu HY Deng YP Zhuang GT Tan BH Huang YZ “Yellow-dragon Wonderful-seed Formula” for hyperuricemia in gout patients with dampness-heat pouring downward pattern: a pilot randomized controlled trial. Trials 2018;19:551. 10.1186/s13063-018-2917-8. 30314508 PMC6186073

[34] Kretova A Wagenaar C Walrabenstein W Vedder D van Schaardenburg D Gerritsen M. Effect of a Whole Food Plant-Based Diet in Patients with Gout: A Pilot Randomized Controlled Trial. Arthritis Rheumatol 2024;76.

[35] Kretova A Wagenaar C Walrabenstein W Vedder D van Schaardenburg D Gerritsen M. Effect of a Whole Food Plant-Based Diet in Patients with Gout: A Pilot Randomized Controlled Trial. Am Med J Rheumatol 2024;1:33–6. 10.33590/rheumatolamj/GEQP5321.

[36] A pilot randomized controlled trial with a one-year extension period on the effect of a whole food plant-based diet for patients with gout | Research with human participants n.d. https://onderzoekmetmensen.nl/en/trial/49186 (accessed June 14, 2025).

[37] Holland R McGill NW. Comprehensive dietary education in treated gout patients does not further improve serum urate. Intern Med J 2015;45:189–94. 10.1111/imj.12661. 25495503

[38] Christensen R Zobbe K Nielsen SM Stamp LK Henriksen M Overgaard AF Weight Loss for Patients With Gout and Concomitant Obesity: A Proof-of-Concept Randomized Trial. Arthritis Rheumatol (Hoboken, NJ) 2024;76:806–12. 10.1002/art.42790. 38169151

[39] Study Details | Weight Loss for Obese Individuals With Gout | ClinicalTrials.gov n.d. https://clinicaltrials.gov/study/NCT03664167 (accessed June 14, 2025).

[40] Singh JA Willig AL Darnell B Green C Morgan S Weiss R Patient-Centered Outcomes and Key Study Procedure Finalization in the Pilot Feasibility Gout Randomized Trial: Comparative Feasibility Study in GOUt, CHerry Extract Versus Diet Modification (Mini-GOUCH). J Clin Rheumatol 2020;26:181–91. 10.1097/RHU.0000000000001018. 30870252 PMC8612296

[41] Juraschek SP Miller ER Wu B White K Charleston J Gelber AC A Randomized Pilot Study of DASH Patterned Groceries on Serum Urate in Individuals with Gout. Nutrients 2021;13:538. 10.3390/nu13020538. 33562216 PMC7914968

[42] Study Details | The Diet Gout Trial | ClinicalTrials.gov n.d. https://clinicaltrials.gov/study/NCT03569020 (accessed June 14, 2025).

[43] Study Details | Pharmacokinetics and Pharmacodynamics of Anthocyanins | ClinicalTrials.gov n.d. https://clinicaltrials.gov/study/NCT03650140 (accessed June 14, 2025).

[44] Study Details | Probiotics for Gout / Hyperuricemia: A Randomized, Intervention, Parallel Controlled, Multicenter Clinical Trial | ClinicalTrials.gov n.d. https://clinicaltrials.gov/study/NCT04199325 (accessed June 14, 2025).

[45] Lamb KL Lynn A Russell J Barker ME. Effect of tart cherry juice on risk of gout attacks: protocol for a randomised controlled trial. BMJ Open 2020;10:e035108. 10.1136/bmjopen-2019-035108. PMC7073821 32179562

[46] Study Details | The Effect of Tart Cherry Juice on Risk of Gout Attacks | ClinicalTrials.gov n.d. https://clinicaltrials.gov/study/NCT03621215 (accessed June 14, 2025).

[47] Singh JA Green C Morgan S Willig AL Darnell B Saag KG A Randomized Internet-Based Pilot Feasibility and Planning Study of Cherry Extract and Diet Modification in Gout. J Clin Rheumatol 2020;26:147–56. 10.1097/RHU.0000000000001004. 32453288 PMC8664374

[48] Ren S Meng F Liu Y Meng Y Tao N Liu R Effects of external application of compound Qingbi granules on acute gouty arthritis with dampness-heat syndrome: a randomized controlled trial. Chin Med 2020;15:117. 10.1186/s13020-020-00398-8. 33292329 PMC7648992

[49] Stamp LK O’Donnell JL Frampton C Drake JM Zhang M Chapman PT. Clinically Insignificant Effect of Supplemental Vitamin C on Serum Urate in Patients With Gout: A Pilot Randomized Controlled Trial. Arthritis Rheum 2013;65:1636–42. 10.1002/art.37925. 23681955

[50] Stamp LK Grainger R Frampton C Drake J Hill CL. Effect of omega-three supplementation on serum urate and gout flares in people with gout; a pilot randomized trial. BMC Rheumatol 2022;6:31. 10.1186/s41927-022-00263-1. 35672866 PMC9175343

[51] Abhishek A Fuller A Nakafero G Zhang W Dumbleton J Hawkey C Feasibility of conducting a randomized, placebo-controlled study assessing whether omega-3 fatty acids prevent gout flares when starting urate-lowering treatment. Rheumatol Adv Pract 2022;6:1–8. 10.1093/RAP/RKAC086. PMC9667976 36407800

[52] Renbin Q Ruizi S Dejiu L Yuanlin C Hongping Y. Treatment of 60 Cases of Gouty Arthritis with Modified Simiao Tang. J Tradit Chinese Med 2008;28:94–7. 10.1016/S0254-6272(08)60023-0. 18652113

[53] Shi X Li G Qian Z Jin Z Song Y. Randomized and controlled clinical study of modified prescriptions of Simiao Pill (四妙 丸) in the treatment of acute gouty arthritis. Chin J Integr Med 2008;14:17–22. 10.1007/s11655-007-9001-7. 18219456

[54] Wei J Xuan-xuan Z Wen-feng T Yan L. Effects of Rebixiao granules on blood uric acid in patients with repeatedly attacking acute gouty arthritis. Chin J Integr Med 2005;11:15–21. 10.1007/BF02835742. 15975301

[55] Zhang J Sun W Gao F Lu J Li K Xu Y Changes of serum uric acid level during acute gout flare and related factors. Front Endocrinol (Lausanne) 2023;14:1077059. 10.3389/fendo.2023.1077059. 36896178 PMC9989260

[56] Halpern R Fuldeore MJ Mody RR Patel PA Mikuls TR. The effect of serum urate on gout flares and their associated costs: an administrative claims analysis. J Clin Rheumatol 2009;15:3–7. 10.1097/RHU.0b013e3181945d2c. 19125135

[57] Schlesinger N Norquist JM Watson DJ. Serum urate during acute gout. J Rheumatol 2009;36:1287–9. 10.3899/jrheum.080938. 19369457

[58] Wang H Zhu B. Basic Theories of Traditional Chinese Medicine 2011:192.

[59] Sun Y Zhao Y Xue SA Chen J. The theory development of traditional Chinese medicine constitution: a review. J Tradit Chinese Med Sci 2018;5:16–28. 10.1016/j.jtcms.2018.02.007.

[60] Emmerson BT. Gout, uric acid and renal disease. Med J Aust 1976;1:403–5. 10.5694/j.1326-5377.1976.tb140703.x. 1272125

[61] Johnson RJ Mandell BF Schlesinger N Mount DB Botson JK Abdellatif AA Controversies and practical management of patients with gout and chronic kidney disease. Kidney Int 2024;106:573–82. 10.1016/j.kint.2024.05.033. 39033815

[62] Liu L Zhu L Liu M Zhao L Yu Y Xue Y Recent Insights Into the Role of Macrophages in Acute Gout. Front Immunol 2022;13:955806. 10.3389/fimmu.2022.955806. 35874765 PMC9304769

[63] So AK Martinon F. Inflammation in gout: mechanisms and therapeutic targets. Nat Rev Rheumatol 2017;13:639–47. 10.1038/nrrheum.2017.155. 28959043

[64] Vedder D Gerritsen M Duvvuri B van Vollenhoven RF Nurmohamed MT Lood C. Neutrophil activation identifies patients with active polyarticular gout. Arthritis Res Ther 2020;22:148. 10.1186/s13075-020-02244-6. 32552822 PMC7304179

[65] Janssen CA Oude Voshaar MAH Ten Klooster PM Vonkeman HE van de Laar MAFJ. Prognostic factors associated with early gout flare recurrence in patients initiating urate-lowering therapy during an acute gout flare. Clin Rheumatol 2019;38:2233–9. 10.1007/s10067-019-04566-6. 31030363

[66] Jiang Y Tu X Liao X He Y Wang S Zhang Q New Inflammatory Marker Associated with Disease Activity in Gouty Arthritis: The Systemic Inflammatory Response Index. J Inflamm Res 2023;16:5565–73. 10.2147/JIR.S432898. 38034046 PMC10683657

[67] Khanna D Ahmed M Yontz D Ginsburg SS Park GS Leonard A The disutility of chronic gout. Qual Life Res 2008;17:815–22. 10.1007/s11136-008-9355-0. 18500578

[68] Watson L Belcher J Nicholls E Chandratre P Blagojevic-Bucknall M Hider S Factors associated with change in health-related quality of life in people with gout: a 3-year prospective cohort study in primary care. Rheumatology (Oxford) 2023;62:2748–56. 10.1093/rheumatology/keac706. 36545704 PMC10393433

[69] Chrysohoou C Skoumas J Pitsavos C Masoura C Siasos G Galiatsatos N Long-term adherence to the Mediterranean diet reduces the prevalence of hyperuricaemia in elderly individuals, without known cardiovascular disease: The Ikaria study. Maturitas 2011;70:58–64. 10.1016/j.maturitas.2011.06.003. 21724344

[70] Cross M Ong KL Culbreth GT Steinmetz JD Cousin E Lenox H Global, regional, and national burden of gout, 1990–2020, and projections to 2050: a systematic analysis of the Global Burden of Disease Study 2021. Lancet Rheumatol 2024;6:e507–17. 10.1016/S2665-9913(24)00117-6. 38996590 PMC11263476

[71] Rai SK Burns LC De Vera MA Haji A Giustini D Choi HK. The economic burden of gout: A systematic review. Semin Arthritis Rheum 2015;45:75–80. 10.1016/j.semarthrit.2015.02.004. 25912932

[72] Sautner J Eichbauer-Sturm G Gruber J Lunzer R Puchner RJ. 2022 update of the Austrian Society of Rheumatology and Rehabilitation nutrition and lifestyle recommendations for patients with gout and hyperuricemia. Wien Klin Wochenschr 2022;134:546–54. 10.1007/s00508-022-02054-7. 35817987 PMC9300548

