# Supplementary material for: Effect of Diet and Dietary Supplements on Gout-Related Outcomes: A Systematic Review of Randomised Controlled Trials
Source: Mediterr J Rheumatol. 2025 Dec 31;36(4):524–38. doi: 10.31138/mjr.010725.era (PMC12869332; doi:10.31138/mjr.010725.era)
Supplement: Supplementary file 1 [file MJR-2025-0187_Supplementary-Tables.pdf]

Supplementary Table 1: PICOS of the study's research question.

**P**opulation	Adult patients (>18 years old) suffering from gout
**I**ntervention	Any diet or dietary supplement
**C**omparison	Any comparison, including usual diet, no intervention, or other diets
**O**utcome	Any disease specific or quality of life related outcome, including specific disease scores, laboratory parameters and patient reported outcomes
**S**tudy design	Randomized controlled trials

Supplementary Table 2: Search syntaxes

PUBMED	((((("gout") OR ("gouty arthritis")) AND ("diet")) OR ("dietary supplement"))) AND (((randomized controlled trial[pt]) OR (controlled clinical trial[pt]) OR (randomized[tiab] OR randomised[tiab]) OR (placebo[tiab]) OR (drug therapy[sh]) OR (randomly[tiab]) OR (trial[tiab]) OR (groups[tiab])) NOT (animals[mh] NOT humans[mh]))
EBSCOhost	"gout" AND "diet"<br>"gout" AND "dietary supplement"<br>"gout" AND "supplement"<br>"gouty arthritis" AND "diet"<br>"gouty arthritis" AND "dietary supplement"<br>"gouty arthritis" AND "supplement"
clinicaltrials.gov	gout AND diet<br>gout AND dietary supplement<br>gout AND supplement<br>gouty arthritis AND diet<br>gouty arthritis AND dietary supplement<br>gouty arthritis AND supplement

Supplementary Table 3: List of excluded studies

First author, publication year	Title	Exclusion reason
Andrés M, 2014	Dietary supplements for chronic gout	Review article
Cândido FG, 2022	Urate-lowering effect of calcium supplementation: Analyses of a randomized controlled trial	Does not assess patients with gout
Chen X, 2024	Comparison of the therapeutic effects of febuxostat combined with a low-purine diet and allopurinol combined with a low-purine diet on the improvement of gout patients	Includes medication treatment intervention
Chen Z, 2024	Effect of low-purine diet on the serum uric acid of gout patients in different clinical subtypes: a prospective cohort study	Does not involve a diet or dietary supplement intervention
Dessein PH, 2000	Beneficial effects of weight loss associated with moderate calorie/carbohydrate restriction, and increased proportional intake of protein and unsaturated fat on serum urate and lipoprotein levels in gout: a pilot study	It is not a randomized controlled trial
Do H, 2024	High vegetable consumption and regular exercise are associated with better quality of life in patients with gout	Prospective cohort study
El-Khodor BF, 2023	A Plant-Based Dietary Supplement Improves Measures of Metabolic Detoxification and the Quality of Life: A Phase II Multicenter Randomized, Blinded, Placebo-Controlled Clinical Trial	Does not assess patients with gout

Gonzalez DE, 2024	Effects of Acute and One-Week Supplementation with Montmorency Tart Cherry Powder on Food-Induced Uremic Response and Markers of Health: A Proof-of-Concept Study	Does not assess patients with gout
Görgülü MB, 2025	Evaluation of treatment compliance in gout patients: a patient-centered study	Not a randomized controlled trial, does not involve a diet or dietary supplement intervention
Habib G, 2014	The Impact of Ramadan Fast on Patients With Gout	Not a randomized controlled trial
Juraschek SP, 2016	Effects of Lowering Glycemic Index of Dietary Carbohydrate on Plasma Uric Acid: The OmniCarb Randomized Clinical Trial	Does not assess patients with gout
Juraschek SP, 2021	Effects of Dietary Patterns on Serum Urate: Results From a Randomized Trial of the Effects of Diet on Hypertension	Does not assess patients with gout
Kai SK, 2024	Adherence to Healthy and Unhealthy Plant-Based Diets and the Risk of Gout	Prospective cohort study
Khanna P, 2020	Development and Pilot Testing of MyGoutCare: A Novel Web-Based Platform to Educate Patients With Gout	Not a randomized controlled trial, does not involve a diet or dietary supplement intervention
Kling J, 2024	Uric Acid Levels, Gout Symptoms Improved With Plant-Based Diet in Pilot Trial | MDedge.	Article in press
Kubomura D, 2016	Tuna extract reduces serum uric acid in gout-free subjects with insignificantly high serum uric acid: A randomized controlled trial	Does not assess patients with gout

Lee S, 2020	Effects of Aster glehni Extract on Serum Uric Acid in Subjects with Mild Hyperuricemia: A Randomized, Placebo-Controlled Trial	Does not assess patients with gout
Liddle J, 2021	‘It’s just a great muddle when it comes to food’: a qualitative exploration of patient decision-making around diet and gout	Does not involve a diet or dietary supplement intervention
Morgan SL, 2020	How do dietary interventions affect serum urate and gout?	Review article
Muhammad KR, 2024	Evaluation of uric acid levels and other biochemical parameters among Gout patients with Ketogenic diet in Erbil province	Case-control observational study
N/A, 2013	Clinical digest. Patients with established gout fail to improve after taking vitamin C supplements.	Article in press
Ponikowska I, 1984	[Results of treatment of patients with diabetes mellitus type 2 associated with obesity and gout]	Article in Polish
Roll S, 2011	Reduction of common cold symptoms by encapsulated juice powder concentrate of fruits and vegetables: a randomised, double-blind, placebo-controlled trial	Does not assess patients with gout
Schiavo L, 2018	Low-Purine Diet Is More Effective Than Normal-Purine Diet in Reducing the Risk of Gouty Attacks After Sleeve Gastrectomy in Patients Suffering of Gout Before Surgery: a Retrospective Study	Retrospective Study
Serlachius A, 2019	Association Between User Engagement of a Mobile Health App for Gout and Improvements in Self-Care Behaviors: Randomized Controlled Trial	Does not involve a diet or dietary supplement intervention
Shi Y, 2016	Quercetin lowers plasma uric acid in pre-hyperuricaemic males: a randomised, double-blinded, placebo-controlled, cross-over trial	Does not assess patients with gout

Singh JA, 2016	A cross-sectional internet-based patient survey of the management strategies for gout	Cross-sectional study, does not assess a diet or dietary supplement intervention
Terkeltaub R, 2025	The Gut Microbiome in Hyperuricemia and Gout.	Review article
Treharne GJ, 2017	Education Preferences of People With Gout: Exploring Differences Between Indigenous and Nonindigenous Peoples from Rural and Urban Locations	Does not involve a diet or dietary supplement intervention
Wang K, 2024	Exploring the anti-gout potential of sunflower receptacles alkaloids: A computational and pharmacological analysis	Not a randomized controlled trial, does not involve a diet or dietary supplement intervention
Wu Y, 2024	Assessment of the efficacy of alkaline water in conjunction with conventional medication for the treatment of chronic gouty arthritis: A randomized controlled study	Retracted article
Yokose C, 2020	Effects of Low-Fat, Mediterranean, or Low-Carbohydrate Weight Loss Diets on Serum Urate and Cardiometabolic Risk Factors: A Secondary Analysis of the Dietary Intervention Randomized Controlled Trial (DIRECT)	Does not assess patients with gout
Zhang M, 2019	Effect of Dietary and Supplemental Omega-3 Polyunsaturated Fatty Acids on Risk of Recurrent Gout Flares	Not a randomized controlled trial

Supplementary Table 4: List of excluded ongoing clinical trials

ClinicalTrials.gov ID	Title	Exclusion reason
NCT01881919	Effect of Quercetin Supplements on Healthy Males: a Four-Week Randomized Cross-Over Trial	Does not assess patients with gout
NCT03636529	Tart Cherry Juice and Markers of Inflammation, CVD, and Diabetes	Does not assess patients with gout
NCT03736551	Intermittent Low Energy Diet in CKD: MIX UP Feasibility Study (MIX-UP)	Does not assess patients with gout
NCT03994731	Study of KRYSTEXXA® (Pegloticase) Plus Methotrexate in Participants With Uncontrolled Gout (MIRROR RCT)	Does not involve a diet or dietary supplement intervention
NCT04875702	Treat-to-Target Serum Urate Versus Treat-to-Avoid Symptoms in Gout (TRUST)	Does not involve a diet or dietary supplement intervention
NCT04938024	Hmong Microbiome ANd Gout, Obesity, Vitamin C (HMANGO-C)	Not a randomized controlled trial
NCT04960527	Acute Effects of Tart Cherry on Uric Acid and Biomarkers of CVD Risk in Healthy Individuals	Does not assess patients with gout
NCT06315543	Effect of Increased Daily Water Intake in Patients With Hyperuricemia	Does not assess patients with gout
NCT06591767	Quercetin as Possible Supportive Therapy for Mild to Moderate Hyperuricemia	Does not assess patients with gout
NCT06652035	Possible Pharmacological Effect of Quercetin in the Management of Hyperuricemia	Does not assess patients with gout

NCT06995339	Comparative Clinical and Biochemical Study Evaluating the Effectiveness of Metformin Versus Febuxostat on Gouty Obese Non-Diabetic Patients	Does not involve a diet or dietary supplement intervention
NCT01363869	Effects of Green Tea on Level of Serum Uric Acid in Healthy Individuals	Does not assess patients with gout
NCT07002762	Multicentre, Double-blind, Randomized, Placebo-controlled Trial of Clinical Efficacy of Burdock Root Extract in Patients With Asymptomatic Hyperuricemia	Does not assess patients with gout

Supplementary table 5. Gout diagnosis and particularities in studies regarding supplements

First author	Gout diagnosis and particularities
Abhishek	ACR/EULAR gout classification criteria (2015); self-report of at least one gout flare in the preceding 12 months; SU 360lmol/l
Dalbeth	American College of Rheumatology diagnostic classification 1977, frequent gout flares at the time of study enrolment (at least two flares in the preceding 4 months)
Ren	ACR/EULAR gout classification criteria (2015)
Renbin	American Society of Rheumatism in 1997 and the Diagnostic Criteria for Gout in Guiding Principles for Clinical Research on New drugs of Chinese Medicine
Schlesinger	Monosodium urate crystal proven gout
Shi	1) An increase in blood uric acid value (the normal value ranges between 89.25-414.76 µmol/L, slight lower in the female); (2) Sudden erythema, swelling, warm sensation, and pain in a single joint (e.g., toe, metatarsus, ankle, knee, elbow, etc.); (3) Sodium urate crystals from the bursal fluid on examination of joint puncture sample; (4) Sodium urate crystal noted in the biopsy of deposits; (5) Perforated defect with proliferative reaction in the border on X-ray examination for the involved joint. The diagnosis can be made by (1) and (2) plus any one of (3)-(5)(1).
Singh	Patient self-reported physician diagnosis of gout, confirmed by contacting participant's healthcare provider who also provided ACR gout classification criteria
Song	Diagnostic standard of gout published in the "Internal Medicine "(4th edition)
Stamp (vitC)	American College of Rheumatology criteria 1977, SU level > 0.36 mmoles/liter (6 mg/dl)
Stamp (fish)	ACR/EULAR gout classification criteria (2015) with serum urate ≥ 0.36 mmol/l
Wang	American College of Rheumatology diagnostic classification 1977
Wei	American College of Rheumatology diagnostic classification 1977, outbreaks twice or more in the past year and a medical history of blood uric acid elevation
Xie	American College of Rheumatology Classification standard for primary gout revised in 1977, with hyperuricemia (blood uric acid [UA] >480mmol/L, without gouty arthritis attack at the time of enrolment
Yu	Participants must have a physician diagnosis of gout* and hyperuricemia (sUA > 420 µmol/L)

ACR: American College of Rheumatology; EULAR: European Alliance of Associations for Rheumatology; SU: serum urate; UA: uric acid.

*Schlesinger N. Diagnosis of Gout: Clinical, Laboratory, and Radiologic Findings. Am J Manag Care. 2005;11:S443–50.

Supplementary table 6. Gout diagnosis and particularities in studies regarding diets

First author	Gout diagnosis and particularities
Christensen	2015 EULAR/ACR gout classification criteria; SU level of at least 5.0 mg/dL; at least one self-reported gout flare in the past year
Holland	ACR 1977 criteria
Juraschek	Self- reported diagnosis of gout and a SU concentration ≥7 mg/dL. Gout was based on self-report in response to the question “Has a physician told you that you have gout?”
Kretova	Diagnosis of gout confirmed by a rheumatologist (first or recurrent episode) based on the ACR 2015 criteria

ACR: American College of Rheumatology; EULAR: European Alliance of Associations for Rheumatology.

Supplementary table 7. Inclusion and exclusion criteria of studies regarding supplements

First author	Inclusion and exclusion criteria
Ahbishek	Inclusion criteria: age >18 yr; meeting the ACR/EULAR gout classification criteria; self-report of at least one gout flare in the preceding 12 months; SU> 360lmol/l; willingness to commence T2T-ULT; and on stable analgesics for 4 wk. Exclusion criteria: ARDs; Lyme disease; psoriasis; asthma or IBD treated with oral immunesuppressing treatment(s); solid organ cancer; dementia; unable to discontinue NSAIDs, oral CSs, omega-3 fatty acids or colchicine; treated with anticoagulants; exposure to systemic CSs in the last month; allergy to omega-3 fatty acids, fish, gelatine, olive oil, soya and unable to take beef products; pregnant/breastfeeding or planning to do so; use of any unlicensed drug within 3 mo before screening or within 5 half-lives of the investigational agent, whichever was longer; haematological or biochemical abnormality, defined as Hb <85 g/l, WBC <3.5 109/l, neut <1.5 109/l, PLT <100 109/l, ALT >1.5 ULN and Cr>2 ULN.
Dalbeth	≥18 yr old; diagnosis of gout (according to the ACR 1977); experiencing frequent gout flares at the time of study enrolment (at least two flares in the preceding 4 mo).11 Exclusion criteria were lactose intolerance and severe renal impairment (eGFR <30 ml/min).
Ren	Inclusion criteria: conforming to the diagnostic criteria of AGA and hyperuricemia; diagnosis of dampness-heat syndrome; age 18–70 yr and any sex; AGA attacked ≥ 1 in the previous yr; alleviation period in previous AGA attacks ≤ 14 days; main observed regions including first metatarsophalangeal joint, dorsum pedis, ankle joint, knee joint, and so forth, and only the most severe joint (target joint) observed and recorded for each participant, with no change during the observation; VAS score in the target joint ≥3; <72 h between the last treatment and the attack; patients who voluntarily participated and signed the written informed consent form. Exclusion criteria: Secondary gout or arthropathy caused by other diseases (e.g., rheumatic arthritis, pyogenic arthritis, traumatic arthritis, senile osteoarthritis, pseudogout, chemotherapy, radiotherapy, chronic lead poisoning, and acute obstructive nephropathy); Chronic intermittent gout or chronic tophaceous gout; > four joints involved in AGA; drugs that affect the metabolism of SU, for example hydrochlorothiazide, furosemide, low-dose aspirin, and drugs that contained the aforementioned components, such as compound reserpine and hydrochlorothiazide; patients who stopped taking CS < 1 mo before enrollment; patients using NSAIDs,

	or other analgesic drugs, or external ointment 24 h before the baseline assessment; severe malformation because of gouty arthropathy or disability resulting from stiffness; pregnancy or lactation; allergic constitution or a history of allergy; Cr exceeding the ULN; AST, ALT > 1.5 ULN; clinically significant arrhythmia; history of alcohol or drug abuse; severe cerebrovascular, renal, liver, or hematopoietic comorbidities, cancer, or mental disorders; participated in other clinical trials in the last 3 mo; referring to the judgment by investigators: some other diseases or situations leading to a lower possibility of recruitment or complicate the enrollment, such as missing visits due to frequent changes in the workplace.
Renbin	Classified Criteria for Gouty Arthritis by American Society of Rheumatism in 1997 and the Diagnostic Criteria for Gout in Guiding Principles for Clinical Research on New drugs of Chinese Medicine issued by the Pharmaceutical Bureau of Health Ministry of China in 1993. Exclusion criteria: functional insufficiency of the heart, lung, liver, and kidney; >70 yrs old; severe deformation and stiffness of joints in late stage; loss of labor ability.
Schlesinger	Patients with monosodium urate crystal proven gout.
Shi	Inclusion criteria: Primary and secondary AGA in the acute attack stage, including primary or recurrent cases; involved joints show obvious symptoms of erythema, swelling, warm sensation and pain; the chalkstone examination shows a positive or negative result. Exclusion Criteria: Intermission period of gout attack; Non-symptomatic hyperuricemic period; Joint swelling and pain induced by rheumatic, rheumatoid, psoriatic arthritis or tuberculosis; Pseudogout induced by an abnormal metabolism of pyrophosphate; severe heart, liver, or renal disease/s; active peptic ulcer and gastrointestinal bleeding.
Singh	Inclusion criteria: US adults ≥ 18 yr; a valid US mailing address and email address; patient self-reported physician diagnosis of gout. Exclusion criteria: self-reported presence of other types of inflammatory arthritis including rheumatoid arthritis or spondyloarthritis; and the current use of cherry extract, juice or concentrate.
Song	Inpatients or outpatients who visited the authors' hospital from 2003 to 2004, all with diagnoses matching the diagnostic standard of gout published in the "Internal Medicine "(4th edition). Exlusion criteria: < 18 yr old; pregnant or lactating women; hypersensitivity or allergies to the test drug; gout secondary to other diseases like nephropathy, myloma, lymph system hyperplasia, hematopathy, having received radio- and/or chemotherapy for malignant tumor, thiazines administration; late stage uremia;

	poor fit to the inclusion standard; taking the test drug irregularly; with incomplete materials or therapeutic effect not to be evaluated.
Stamp (VitC)	Patients with gout, whose diagnosis was defined according to ACR 1977 and with an SU level 0.36 mmoles/liter (6 mg/dl). Patients taking over-the-counter vitamin supplements were excluded.
Stamp fish	≥18 yr old; gout as defined by the 2015 Gout Classification Criteria; SU ≥ 0.36 mmol/l and either on a stable dose of allopurinol for at least one month or on no urate lowering therapy. Exclusion criteria: history of intolerance or allergy to omega three fatty acid supplements or an allergy to fish or shellfish; implantable defibrillator or receiving warfarin or dabigatran.
Wang	Inclusion criteria: Adults with a new clinical diagnosis of gout according to the 1977 ACR criteria and an onset of the disease duration of less than 48 hours. Exclusion criteria: secondary gout, such as rheumatoid arthritis, septic arthritis, traumatic arthritis, etc.; in the intermittent period or with tophi; taking diuretics, pyrazinamide, aspirin, etc.; pregnancy, breast-feeding women; cardiovascular and cerebral vascular disease, severe trauma or had undergone surgery; severe infections; hepatobiliary disease or AST, ALT >2 times ULN; Cr > ULN; severe chronic gastrointestinal disease; hematological diseases or endocrine system diseases; undergoing cancer treatment; receiving CS; allergic to those known ingredients in the Chuanhu anti-gout mixture.
Wei	ACR 1977 criteria; outbreaks twice or more in the past yr; SU elevation; 18-75 yr old, with sex disregarded. The syndrome differentiation standard complies with the Gout Diagnosis and Treatment Effects Standard of TCM Professional Standard of P. R. C (1995) with secondary gout excluded.
Xie	Inclusion criteria: male patients; 18-60 yr, with hyperuricemia (SU >480mmol/L), meeting ACR 1977 criteria; without gouty arthritis attack at the time of enrolment; willing to participate in the trial and sign informed consent certificates. Exclusion criteria: acute gouty arthritis at the baseline; administered a uricosuric medicine within 2 wk; secondary gout, with Cr >133mmol/L or urinary calculi; serious organ dysfunction; mental illness; cancer, and body mass index>50kg/m2; alcoholism.
Yu	Physician diagnosis of gout; hyperuricemia (SU> 420 µmol/L); 18– 70 yr. If the patient had already received treatment for gout, he/she had to undergo a two-week washout period; only those whose SU remained >420 µmol/L after the washout period could be included. Only patients differentiated as dampness-heat pouring downward pattern were included. The dampness-heat pouring downward pattern was confirmed by clinical symptoms and signs manifested as red, swollen, hot, and painful

acute joint arthritis, red tongue, yellow and greasy tongue coating, and smooth pulse. The pattern must be differentiated by at least two trained doctors of TCM. Exclusion criteria: pregnancy or lactation; allergic constitution, or an allergic history to test TCM or allopurinol; Cr> 1.5 mg/dL; elevated values of ALT twice as high as the ULN; severe deformity or stiffness of gouty arthropathy resulting in disability; arrhythmia of clinical significance; history of alcohol abuse; severe cerebrovascular, kidney, liver, or hematopoietic system comorbidities, cancer or mental disorders; taken concurrent hypouricemic medications, azathioprine, 6-mercaptopurine, medications containing aspirin (>325 mg) or salicylate; or had participated in other clinical trials within the past three months.

ACR: American College of Rheumatology; AGA: Acute gouty arthritis; ALT: Alanine aminotransferase; ARD: Autoimmune rheumatic diseases; AST: Aspartate aminotransferase; Cr: creatinine; CS: Corticosteroids; eGFR: estimated glomerular filtration rate; Hb: haemoglobin; mo: months; neut: neutrophils; NSAIDS: Non steroid anti-inflammatory drugs; PLT: Platelets; SU: Serum urate; T2T-ULT: Treat to target urate lowering therapy; ULN: upper limit of normal; WBC: white blood cells; wk: weeks; yr: year.

Supplementary table 8. Concurrent gout treatment of studies regarding dietary supplements

First author	Gout treatment and anti-inflammatory/urate lowering medication
Abhishek	Dose up-titration visits occurred at 2- to 3-weekly intervals. Participants already on ULT at study entry had their dose optimized aiming for SU <300 µmol/l. Gout flares were treated with prednisolone (enteric coated) 30 mg/day with a proton pump inhibitor for 1 week. If CSs were contraindicated or if participants preferred not to take CSs, they were prescribed naproxen 500 mg twice a day with a proton pump inhibitor for 1week. Participants were allowed to take analgesics as required during the gout flare.
Delboth	The protocol did not specify any other changes to gout management during the study, and gout flares occurring during the study were treated according to the discretion of the patient's usual doctor. Some patients were on allopurinol, colchicine, prednisone or NSAIDs but there were no differences between groups.
Ren	Participants in all groups received Western medicine basic treatment, including low-purine diet, drinking water more than 2000 mL/days, three times loxoprofen (60 mg each time) and NAHCO3 (1 g each time) per day.
Renbin	Other analgesics and the drugs interfering with the UA were contraindicated during the treatment.
Schlesinger	Patients continued use of allopurinol, prophylactic colchicine or NSAIDs if they have been taking these drugs prior to initiating the study.
Shi	ndomethacin and benzobromarone were part of the control group intervention. Allopurinol and colchicine are not mentioned in the study.
Singh	Some patients were taking allopurinol, febuxostat or probenecid (33% in intervention group and 42% in diet group; difference ns). A quarter of patients took colchicine.
Song	All patients were put on low purine diet with a large quantity of water intake to maintain a daily urine amount up to 2 000-3 000 mL. The pH value of urine was maintained within 6.0-6.5 by oral intake of 3 g of sodium bicarbonate per day (product of Tianjin Lisheng Pharmaceutical Co., Ltd., batch No. 020419).

Stamp (VitC)	Allopurinol was started at a dose of 50 mg or 100 mg, or the dose was increased by these increments, at the discretion of the physician, depending on each patient's renal function and comorbidities. The dose of allopurinol was further increased at 4 weeks if the patient had not achieved the target SU level of 0.36 mmoles/liter (6 mg/dl) as per standard clinical practice.
Stamp (fish)	Patients were either on a stable dose of allopurinol for at least one month or on no urate lowering therapy were recruited. For those on allopurinol the dose remained stable during the entire study period.
Wang	No steroids. In addition, all the patients were required to rest remaining in bed and avoiding lifting the affected limb, and avoid overtiring, tension and cold; were given a low purine diet, abstaining from smoking, wine, animal giblets, fish, shrimp and unfermented soybean foods; were required to drink enough water, 2000-3000 ml daily; received sodium bicarbonate 1.0 g three times daily orally to alkalinize the urine; received etoricoxib 60 mg daily orally for 10 days to relieve pain in the affected joints
Wei	NSAIDs were part of the control group. Allopurinol and colchicine are not mentioned in the study
Xie	At baseline, every patient was instructed to reduce their purine- rich food intake and to drink >2 L of water a day. Exclusion criteria included administration of uricosuric medication within 2wks. Treatment with other gout therapies during the study period rendered the participant as an invalid case.
Yu	If the patient had already received treatment for gout, he/she had to undergo a two-week washout period; only those whose sUA remained >420 µmol/L after the washout period could be included. Patients were excluded if they were taking concurrent hypouricemic medications. All patients were advised not to consume purine-rich diets.

CS: corticosteroids; NSAIDs: non-steroidal anti-inflammatory drugs; SUA: serum uric acid.

Supplementary table 9. Inclusion and exclusion criteria of studies regarding diets

First author	Inclusion and exclusion criteria
Christensen	>18 yr old; obesity (BMI > 30); 2015 EULAR/ACR criteria; SU level of at least 5.0 mg/dL; at least one self-reported gout flare in the past year. Exclusion criteria: Other inflammatory diseases; cancer; took part in other trials, including pharmacological studies or weight loss trials
Holland	>18 yr old; history of gout as per ACR criteria; on a stable dose of urate lowering therapy at target (SU<0.36 mmol/L). Exclusion criteria: unable to communicate in English (both verbal and written, to standardize information)
Juraschek	Inclusion criteria: ≥18 yr, self- reported diagnosis of gout; SU concentration ≥7 mg/dL; Exclusion criteria: active use of or plans for urate lowering therapy, excessive alcohol use, stage 4 or 5 chronic kidney disease, unstable medication use (steroid, lipid-lowering, or antihypertensive agents), active prescriptions of warfarin or insulin, major gastrointestinal conditions affecting food absorption, or inability to store food at home.
Kretova	Gout, hyperuricemia (males ≥0,42 mmol/L and females ≥0,36 mmol/L), abdominal obesity (waist circumference of ≥102 cm for males and ≥88 cm for females), and not receiving urate-lowering therapy. Exclusion criteria : Concurrent presence of other forms of inflammatory joint disease than gout; intractable gout due to side effects or contraindications for standard flare treatment (NSAIDs, colchicine and corticosteroids); current use of urate lowering therapy or use of urate lowering therapy in the last 30 days; indication for urate lowering therapy according national guidelines (NVR gout 2013) including 2 or more flares in one year, tophaceous gout or history of urate urolithiasis unless agreement between patient and treating rheumatologist led to the decision to postpone the start of ULT for the duration of at least the first 4 months of the study; pregnancy; insufficient comprehension of Dutch language; already following a (near-)vegan diet; in case of smoking, unwillingness to stop smoking for at least the duration of the study; low e-health competencies (lowest proficiency according to Pharos quick scan); inability to be scheduled for counselling and measurement visits; psychiatric disease; no informed consent

ACR: American College of Rheumatology; BMI: body mass index (kg/m^2); EULAR: European Alliance of Associations for Rheumatology; kg: kilogram; m:meter; ULT: urate-lowering therapy.

Supplementary table 10. Concurrent gout treatment of studies regarding diet

First author	Gout treatment and anti-inflammatory/urate lowering medication
Christensen	Participants were allowed to use ULT, but they were not allowed to change ULT type or dose during the trial
Holland	Stable dose of urate lowering therapy at target (serum urate <0.36 mmol/L),
Juraschek	Exclusion criteria included active use of or plans for urate lowering therapy, unstable medication use (steroids). Colchicine and NSAIDS in both groups were used
Kretova	Participants were allowed to use their usual gout flare medication during a gout flare

NSAIDs: non-steroidal anti-inflammatory drugs; ULT: urate-lowering therapy.
