# Supplementary material for: Effect of Diet and Dietary Supplements on Gout-Related Outcomes: A Systematic Review of Randomised Controlled Trials
Source: Mediterr J Rheumatol. 2025 Dec 31;36(4):524–38. doi: 10.31138/mjr.010725.era (PMC12869332; doi:10.31138/mjr.010725.era)
Supplement: Supplementary file 2 [file MJR-2025-0187_Supplementary-Tables.docx]

Supplementary Table 1: PICOS of the study’s research question.

| **P**opulation | Adult patients (>18 years old) suffering from gout |
| --- | --- |
| **I**ntervention | Any diet or dietary supplement |
| **C**omparison | Any comparison, including usual diet, no intervention, or other diets |
| **O**utcome | Any disease specific or quality of life related outcome, including specific disease scores, laboratory parameters and patient reported outcomes |
| **S**tudy design | Randomized controlled trials |

Supplementary Table 2: Search syntaxes

| PUBMED | ((((("gout") OR ("gouty arthritis")) AND ("diet")) OR ("dietary supplement"))) AND (((randomized controlled trial[pt]) OR (controlled clinical trial[pt]) OR (randomized[tiab] OR randomised[tiab]) OR (placebo[tiab]) OR (drug therapy[sh]) OR (randomly[tiab]) OR (trial[tiab]) OR (groups[tiab])) NOT (animals[mh] NOT humans[mh])) |
| --- | --- |
| EBSCOhost | "gout" AND "diet"  "gout" AND "dietary supplement"  "gout" AND "supplement"  "gouty arthritis" AND "diet"  "gouty arthritis" AND "dietary supplement"  "gouty arthritis" AND "supplement" |
| clinicaltrials.gov | gout AND diet  gout AND dietary supplement  gout AND supplement  gouty arthritis AND diet  gouty arthritis AND dietary supplement  gouty arthritis AND supplement |

Supplementary Table 3: List of excluded studies

| First author, publication year | Title | Exclusion reason |
| --- | --- | --- |
| Andrés M, 2014 | Dietary supplements for chronic gout | Review article |
| Cândido FG, 2022 | Urate-lowering effect of calcium supplementation: Analyses of a randomized controlled trial | Does not assess patients with gout |
| Chen X, 2024 | Comparison of the therapeutic effects of febuxostat combined with a low-purine diet and allopurinol combined with a low-purine diet on the improvement of gout patients | Includes medication treatment intervention |
| Chen Z, 2024 | Effect of low-purine diet on the serum uric acid of gout patients in different clinical subtypes: a prospective cohort study | Does not involve a diet or dietary supplement intervention |
| Dessein PH, 2000 | Beneficial effects of weight loss associated with moderate calorie/carbohydrate restriction, and increased proportional intake of protein and unsaturated fat on serum urate and lipoprotein levels in gout: a pilot study | It is not a randomized controlled trial |
| Do H, 2024 | High vegetable consumption and regular exercise are associated with better quality of life in patients with gout | Prospective cohort study |
| El-Khodor BF, 2023 | A Plant-Based Dietary Supplement Improves Measures of Metabolic Detoxification and the Quality of Life: A Phase II Multicenter Randomized, Blinded, Placebo-Controlled Clinical Trial | Does not assess patients with gout |
| Gonzalez DE, 2024 | Effects of Acute and One-Week Supplementation with Montmorency Tart Cherry Powder on Food-Induced Uremic Response and Markers of Health: A Proof-of-Concept Study | Does not assess patients with gout |
| Görgülü MB, 2025 | Evaluation of treatment compliance in gout patients: a patient-centered study | Not a randomized controlled trial, does not involve a diet or dietary supplement intervention |
| Habib G, 2014 | The Impact of Ramadan Fast on Patients With Gout | Not a randomized controlled trial |
| Juraschek SP, 2016 | Effects of Lowering Glycemic Index of Dietary Carbohydrate on Plasma Uric Acid: The OmniCarb Randomized Clinical Trial | Does not assess patients with gout |
| Juraschek SP, 2021 | Effects of Dietary Patterns on Serum Urate: Results From a Randomized Trial of the Effects of Diet on Hypertension | Does not assess patients with gout |
| Kai SK, 2024 | Adherence to Healthy and Unhealthy Plant-Based Diets and the Risk of Gout | Prospective cohort study |
| Khanna P, 2020 | Development and Pilot Testing of MyGoutCare: A Novel Web-Based Platform to Educate Patients With Gout | Not a randomized controlled trial, does not involve a diet or dietary supplement intervention |
| Kling J, 2024 | Uric Acid Levels, Gout Symptoms Improved With Plant-Based Diet in Pilot Trial \| MDedge. | Article in press |
| Kubomura D, 2016 | Tuna extract reduces serum uric acid in gout-free subjects with insignificantly high serum uric acid: A randomized controlled trial | Does not assess patients with gout |
| Lee S, 2020 | Effects of Aster glehni Extract on Serum Uric Acid in Subjects with Mild Hyperuricemia: A Randomized, Placebo-Controlled Trial | Does not assess patients with gout |
| Liddle J, 2021 | ‘It’s just a great muddle when it comes to food’: a qualitative exploration of patient decision-making around diet and gout | Does not involve a diet or dietary supplement intervention |
| Morgan SL, 2020 | How do dietary interventions affect serum urate and gout? | Review article |
| Muhammad KR, 2024 | Evaluation of uric acid levels and other biochemical parameters among Gout patients with Ketogenic diet in Erbil province | Case-control observational study |
| N/A, 2013 | Clinical digest. Patients with established gout fail to improve after taking vitamin C supplements. | Article in press |
| Ponikowska I, 1984 | [Results of treatment of patients with diabetes mellitus type 2 associated with obesity and gout] | Article in Polish |
| Roll S, 2011 | Reduction of common cold symptoms by encapsulated juice powder concentrate of fruits and vegetables: a randomised, double-blind, placebo-controlled trial | Does not assess patients with gout |
| Schiavo L, 2018 | Low-Purine Diet Is More Effective Than Normal-Purine Diet in Reducing the Risk of Gouty Attacks After Sleeve Gastrectomy in Patients Suffering of Gout Before Surgery: a Retrospective Study | Retrospective Study |
| Serlachius A, 2019 | Association Between User Engagement of a Mobile Health App for Gout and Improvements in Self-Care Behaviors: Randomized Controlled Trial | Does not involve a diet or dietary supplement intervention |
| Shi Y, 2016 | Quercetin lowers plasma uric acid in pre-hyperuricaemic males: a randomised, double-blinded, placebo-controlled, cross-over trial | Does not assess patients with gout |
| Singh JA, 2016 | A cross-sectional internet-based patient survey of the management strategies for gout | Cross-sectional study, does not assess a diet or dietary supplement intervention |
| Terkeltaub R, 2025 | The Gut Microbiome in Hyperuricemia and Gout. | Review article |
| Treharne GJ, 2017 | Education Preferences of People With Gout: Exploring Differences Between Indigenous and Nonindigenous Peoples from Rural and Urban Locations | Does not involve a diet or dietary supplement intervention |
| Wang K, 2024 | Exploring the anti-gout potential of sunflower receptacles alkaloids: A computational and pharmacological analysis | Not a randomized controlled trial, does not involve a diet or dietary supplement intervention |
| Wu Y, 2024 | Assessment of the efficacy of alkaline water in conjunction with conventional medication for the treatment of chronic gouty arthritis: A randomized controlled study | Retracted article |
| Yokose C, 2020 | Effects of Low-Fat, Mediterranean, or Low-Carbohydrate Weight Loss Diets on Serum Urate and Cardiometabolic Risk Factors: A Secondary Analysis of the Dietary Intervention Randomized Controlled Trial (DIRECT) | Does not assess patients with gout |
| Zhang M, 2019 | Effect of Dietary and Supplemental Omega-3 Polyunsaturated Fatty Acids on Risk of Recurrent Gout Flares | Not a randomized controlled trial |

Supplementary Table 4: List of excluded ongoing clinical trials

| ClinicalTrials.gov ID | Title | Exclusion reason |
| --- | --- | --- |
| NCT01881919 | Effect of Quercetin Supplements on Healthy Males: a Four-Week Randomized Cross-Over Trial | Does not assess patients with gout |
| NCT03636529 | Tart Cherry Juice and Markers of Inflammation, CVD, and Diabetes | Does not assess patients with gout |
| NCT03736551 | Intermittent Low Energy Diet in CKD: MIX UP Feasibility Study (MIX-UP) | Does not assess patients with gout |
| NCT03994731 | Study of KRYSTEXXA® (Pegloticase) Plus Methotrexate in Participants With Uncontrolled Gout (MIRROR RCT) | Does not involve a diet or dietary supplement intervention |
| NCT04875702 | Treat-to-Target Serum Urate Versus Treat-to-Avoid Symptoms in Gout (TRUST) | Does not involve a diet or dietary supplement intervention |
| NCT04938024 | Hmong Microbiome ANd Gout, Obesity, Vitamin C (HMANGO-C) | Not a randomized controlled trial |
| NCT04960527 | Acute Effects of Tart Cherry on Uric Acid and Biomarkers of CVD Risk in Healthy Individuals | Does not assess patients with gout |
| NCT06315543 | Effect of Increased Daily Water Intake in Patients With Hyperuricemia | Does not assess patients with gout |
| NCT06591767 | Quercetin as Possible Supportive Therapy for Mild to Moderate Hyperuricemia | Does not assess patients with gout |
| NCT06652035 | Possible Pharmacological Effect of Quercetin in the Management of Hyperuricemia | Does not assess patients with gout |
| NCT06995339 | Comparative Clinical and Biochemical Study Evaluating the Effectiveness of Metformin Versus Febuxostat on Gouty Obese Non-Diabetic Patients | Does not involve a diet or dietary supplement intervention |
| NCT01363869 | Effects of Green Tea on Level of Serum Uric Acid in Healthy Individuals | Does not assess patients with gout |
| NCT07002762 | Multicentre, Double-blind, Randomized, Placebo-controlled Trial of Clinical Efficacy of Burdock Root Extract in Patients With Asymptomatic Hyperuricemia | Does not assess patients with gout |

*Supplementary table 5. Gout diagnosis and particularities in studies regarding supplements*

| First author | Gout diagnosis and particularities |
| --- | --- |
| Abhishek | ACR/EULAR gout classification criteria (2015); self-report of at least one gout flare in the preceding 12 months; SU 360lmol/l |
| Dalbeth | American College of Rheumatology diagnostic classification 1977, frequent gout flares at the time of study enrolment (at least two flares in the preceding 4 months) |
| Ren | ACR/EULAR gout classification criteria (2015) |
| Renbin | American Society of Rheumatism in 1997 and the Diagnostic Criteria for Gout in Guiding Principles for Clinical Research on New drugs of Chinese Medicine |
| Schlesinger | Monosodium urate crystal proven gout |
| Shi | 1) An increase in blood uric acid value (the normal value ranges between 89.25-414.76 μmol/L, slight lower in the female); (2) Sudden erythema, swelling, warm sensation, and pain in a single joint (e.g., toe, metatarsus, ankle, knee, elbow, etc.); (3) Sodium urate crystals from the bursal fluid on examination of joint puncture sample; (4) Sodium urate crystal noted in the biopsy of deposits; (5) Perforated defect with proliferative reaction in the border on X-ray examination for the involved joint. The diagnosis can be made by (1) and (2) plus any one of (3)-(5)(1). |
| Singh | Patient self-reported physician diagnosis of gout, confirmed by contacting participant’s healthcare provider who also provided ACR gout classification criteria |
| Song | Diagnostic standard of gout published in the "Internal Medicine "(4th edition) |
| Stamp (vitC) | American College of Rheumatology criteria 1977, SU level > 0.36 mmoles/liter (6 mg/dl) |
| Stamp (fish) | ACR/EULAR gout classification criteria (2015) with serum urate ≥ 0.36 mmol/l |
| Wang | American College of Rheumatology diagnostic classification 1977 |
| Wei | American College of Rheumatology diagnostic classification 1977, outbreaks twice or more in the past year and a medical history of blood uric acid elevation |
| Xie | American College of Rheumatology Classification standard for primary gout revised in 1977, with hyperuricemia (blood uric acid [UA] >480mmol/L, without gouty arthritis attack at the time of enrolment |
| Yu | Participants must have a physician diagnosis of gout* and hyperuricemia (sUA > 420 μmol/L) |

ACR: American College of Rheumatology; EULAR: European Alliance of Associations for Rheumatology; SU: serum urate; UA: uric acid.

*Schlesinger N. Diagnosis of Gout: Clinical, Laboratory, and Radiologic Findings. Am J Manag Care. 2005;11:S443–50.

*Supplementary table 6. Gout diagnosis and particularities in studies regarding diets*

| First author | Gout diagnosis and particularities |
| --- | --- |
| Christensen | 2015 EULAR/ACR gout classification criteria; SU level of at least 5.0 mg/dL; at least one self-reported gout flare in the past year |
| Holland | ACR 1977 criteria |
| Juraschek | Self- reported diagnosis of gout and a SU concentration ≥7 mg/dL. Gout was based on self- report in response to the question “Has a physician told you that you have gout?” |
| Kretova | Diagnosis of gout confirmed by a rheumatologist (first or recurrent episode) based on the ACR 2015 criteria |

ACR: American College of Rheumatology; EULAR: European Alliance of Associations for Rheumatology*.*

*Supplementary table 7. Inclusion and exclusion criteria of studies regarding supplements*

| First author | Inclusion and exclusion criteria |
| --- | --- |
| Ahbishek | Inclusion criteria: age >18 yr; meeting the ACR/EULAR gout classification criteria; self-report of at least one gout flare in the preceding 12 months; SU> 360lmol/l; willingness to commence T2T-ULT; and on stable analgesics for 4 wk. Exclusion criteria: ARDs; Lyme disease; psoriasis; asthma or IBD treated with oral immunesuppressing treatment(s); solid organ cancer; dementia; unable to discontinue NSAIDs, oral CSs, omega-3 fatty acids or colchicine; treated with anticoagulants; exposure to systemic CSs in the last month; allergy to omega-3 fatty acids, fish, gelatine, olive oil, soya and unable to take beef products; pregnant/breastfeeding or planning to do so; use of any unlicensed drug within 3 mo before screening or within 5 half-lives of the investigational agent, whichever was longer; haematological or biochemical abnormality, defined as Hb <85 g/l, WBC <3.5 109/l, neut <1.5 109/l, PLT <100 109/l, ALT >1.5 ULN and Cr>2 ULN. |
| Dalbeth | ≥18 yr old; diagnosis of gout (according to the ACR 1977); experiencing frequent gout flares at the time of study enrolment (at least two flares in the preceding 4 mo).11 Exclusion criteria were lactose intolerance and severe renal impairment (eGFR <30 ml/min). |
| Ren | Inclusion criteria: conforming to the diagnostic criteria of AGA and hyperuricemia; diagnosis of dampness-heat syndrome; age 18–70 yr and any sex; AGA attacked ≥ 1 in the previous yr; alleviation period in previous AGA attacks ≤ 14 days; main observed regions including first metatarsophalangeal joint, dorsum pedis, ankle joint, knee joint, and so forth, and only the most severe joint (target joint) observed and recorded for each participant, with no change during the observation; VAS score in the target joint ≥3; <72 h between the last treatment and the attack; patients who voluntarily participated and signed the written informed consent form. Exclusion criteria: Secondary gout or arthropathy caused by other diseases (e.g., rheumatic arthritis, pyogenic arthritis, traumatic arthritis, senile osteoarthritis, pseudogout, chemotherapy, radiotherapy, chronic lead poisoning, and acute obstructive nephropathy); Chronic intermittent gout or chronic tophaceous gout; > four joints involved in AGA; drugs that affect the metabolism of SU, for example hydrochlorothiazide, furosemide, low-dose aspirin, and drugs that contained the aforementioned components, such as compound reserpine and hydrochlorothiazide; patients who stopped taking CS < 1 mo before enrollment; patients using NSAIDs, or other analgesic drugs, or external ointment 24 h before the baseline assessment; severe malformation because of gouty arthropathy or disability resulting from stiffness; pregnancy or lactation; allergic constitution or a history of allergy; Cr exceeding the ULN; AST, ALT > 1.5 ULN; clinically significant arrhythmia; history of alcohol or drug abuse; severe cerebrovascular, renal, liver, or hematopoietic comorbidities, cancer, or mental disorders; participated in other clinical trials in the last 3 mo; referring to the judgment by investigators: some other diseases or situations leading to a lower possibility of recruitment or complicate the enrollment, such as missing visits due to frequent changes in the workplace. |
| Renbin | Classified Criteria for Gouty Arthritis by American Society of Rheumatism in 1997 and the Diagnostic Criteria for Gout in Guiding Principles for Clinical Research on New drugs of Chinese Medicine issued by the Pharmaceutical Bureau of Health Ministry of China in 1993. Exclusion criteria: functional insufficiency of the heart, lung, liver, and kidney; >70 yrs old; severe deformation and stiffness of joints in late stage; loss of labor ability. |
| Schlesinger | Patients with monosodium urate crystal proven gout. |
| Shi | Inclusion criteria: Primary and secondary AGA in the acute attack stage, including primary or recurrent cases; involved joints show obvious symptoms of erythema, swelling, warm sensation and pain; the chalkstone examination shows a positive or negative result. Exclusion Criteria: Intermission period of gout attack; Non-symptomatic hyperuricemic period; Joint swelling and pain induced by rheumatic, rheumatoid, psoriatic arthritis or tuberculosis; Pseudogout induced by an abnormal metabolism of pyrophosphate; severe heart, liver, or renal disease/s; active peptic ulcer and gastrointestinal bleeding. |
| Singh | Inclusion criteria: US adults ≥ 18 yr; a valid US mailing address and email address; patient self-reported physician diagnosis of gout. Exclusion criteria: self-reported presence of other types of inflammatory arthritis including rheumatoid arthritis or spondyloarthritis; and the current use of cherry extract, juice or concentrate. |
| Song | Inpatients or outpatients who visited the authors' hospital from 2003 to 2004, all with diagnoses matching the diagnostic standard of gout published in the "Internal Medicine "(4th edition). Exlusion criteria: < 18 yr old; pregnant or lactating women; hypersensitivity or allergies to the test drug; gout secondary to other diseases like nephropathy, myloma, lymph system hyperplasia, hematopathy, having received radio- and/or chemotherapy for malignant tumor, thiazines administration; late stage uremia; poor fit to the inclusion standard; taking the test drug irregularly; with incomplete materials or therapeutic effect not to be evaluated. |
| Stamp (VitC) | Patients with gout, whose diagnosis was defined according to ACR 1977 and with an SU level 0.36 mmoles/liter (6 mg/dl). Patients taking over-the-counter vitamin supplements were excluded. |
| Stamp fish | ≥18 yr old; gout as defined by the 2015 Gout Classification Criteria; SU ≥ 0.36 mmol/l and either on a stable dose of allopurinol for at least one month or on no urate lowering therapy. Exclusion criteria: history of intolerance or allergy to omega three fatty acid supplements or an allergy to fish or shellfish; implantable defibrillator or receiving warfarin or dabigatran. |
| Wang | Inclusion criteria: Adults with a new clinical diagnosis of gout according to the 1977 ACR criteria and an onset of the disease duration of less than 48 hours. Exclusion criteria: secondary gout, such as rheumatoid arthritis, septic arthritis, traumatic arthritis, etc.; in the intermittent period or with tophi; taking diuretics, pyrazinamide, aspirin, etc.; pregnancy, breast-feeding women; cardiovascular and cerebral vascular disease, severe trauma or had undergone surgery; severe infections; hepatobiliary disease or AST, ALT >2 times ULN; Cr > ULN; severe chronic gastrointestinal disease; hematological diseases or endocrine system diseases; undergoing cancer treatment; receiving CS; allergic to those known ingredients in the Chuanhu anti-gout mixture. |
| Wei | ACR 1977 criteria; outbreaks twice or more in the past yr; SU elevation; 18-75 yr old, with sex disregarded. The syndrome differentiation standard complies with the Gout Diagnosis and Treatment Effects Standard of TCM Professional Standard of P. R. C (1995) with secondary gout excluded. |
| Xie | Inclusion criteria: male patients; 18 -60 yr, with hyperuricemia (SU >480mmol/L), meeting ACR 1977 criteria; without gouty arthritis attack at the time of enrolment; willing to participate in the trial and sign informed consent certificates. Exclusion criteria: acute gouty arthritis at the baseline; administered a uricosuric medicine within 2 wk; secondary gout, with Cr >133mmol/L or urinary calculi; serious organ dysfunction; mental illness; cancer, and body mass index>50kg/m2; alcoholism. |
| Yu | Physician diagnosis of gout; hyperuricemia (SU> 420 μmol/L); 18– 70 yr. If the patient had already received treatment for gout, he/she had to undergo a two-week washout period; only those whose SU remained >420 μmol/L after the washout period could be included. Only patients differentiated as dampness-heat pouring downward pattern were included. The dampness-heat pouring downward pattern was confirmed by clinical symptoms and signs manifested as red, swollen, hot, and painful acute joint arthritis, red tongue, yellow and greasy tongue coating, and smooth pulse. The pattern must be differentiated by at least two trained doctors of TCM. Exclusion criteria: pregnancy or lactation; allergic constitution, or an allergic history to test TCM or allopurinol; Cr> 1.5 mg/dL; elevated values of ALT twice as high as the ULN; severe deformity or stiffness of gouty arthropathy resulting in disability; arrhythmia of clinical significance; history of alcohol abuse; severe cerebrovascular, kidney, liver, or hematopoietic system comorbidities, cancer or mental disorders; taken concurrent hypouricemic medications, azathioprine, 6-mercaptopurine, medications containing aspirin (>325 mg) or salicylate; or had participated in other clinical trials within the past three months. |

ACR: American College of Rheumatology; AGA: Acute gouty arthritis; ALT: Alanine aminotransferase; ARD: Autoimmune rheumatic diseases; AST: Aspartate aminotransferase; Cr: creatinine; CS: Corticosteroids; eGFR: estimated glomerular filtration rate; Hb: haemoglobin; mo: months; neut: neutrophils; NSAIDS: Non steroid anti-inflammatory drugs; PLT: Platelets; SU: Serum urate; T2T-ULT: Treat to target urate lowering therapy; ULN: upper limit of normal; WBC: white blood cells; wk: weeks; yr: year.

*Supplementary table 8. Concurrent gout treatment of studies regarding dietary supplements*

| First author | Gout treatment and anti-inflammatory/urate lowering medication |
| --- | --- |
| Abhishek | Dose up-titration visits occurred at 2- to 3-weekly intervals. Participants already on ULT at study entry had their dose optimized aiming for SU <300 μmol/l. Gout flares were treated with prednisolone (enteric coated) 30 mg/day with a proton pump inhibitor for 1 week. If CSs were contraindicated or if participants preferred not to take CSs, they were prescribed naproxen 500 mg twice a day with a proton pump inhibitor for 1week. Participants were allowed to take analgesics as required during the gout flare. |
| Delboth | The protocol did not specify any other changes to gout management during the study, and gout flares occurring during the study were treated according to the discretion of the patient’s usual doctor. Some patients were on allopurinol, colchicine, prednisone or NSAIDs but there were no differences between groups. |
| Ren | Participants in all groups received Western medicine basic treatment, including low-purine diet, drinking water more than 2000 mL/days, three times loxoprofen (60 mg each time) and NAHCO3 (1 g each time) per day. |
| Renbin | Other analgesics and the drugs interfering with the UA were contraindicated during the treatment. |
| Schlesinger | Patients continued use of allopurinol, prophylactic colchicine or NSAIDs if they have been taking these drugs prior to initiating the study. |
| Shi | ndomethacin and benzobromarone were part of the control group intervention. Allopurinol and colchicine are not mentioned in the study. |
| Singh | Some patients were taking allopurinol, febuxostat or probenecid (33% in intervention group and 42% in diet group; difference ns). A quarter of patients took colchicine. |
| Song | All patients were put on low purine diet with a large quantity of water intake to maintain a daily urine amount up to 2 000-3 000 mL. The pH value of urine was maintained within 6.0-6.5 by oral intake of 3 g of sodium bicarbonate per day (product of Tianjin Lisheng Pharmaceutical Co., Ltd., batch No. 020419). |
| Stamp (VitC) | Allopurinol was started at a dose of 50 mg or 100 mg, or the dose was increased by these increments, at the discretion of the physician, depending on each patient’s renal function and comorbidities. The dose of allopurinol was further increased at 4 weeks if the patient had not achieved the target SU level of 0.36 mmoles/liter (6 mg/dl) as per standard clinical practice. |
| Stamp (fish) | Patients were either on a stable dose of allopurinol for at least one month or on no urate lowering therapy were recruited. For those on allopurinol the dose remained stable during the entire study period. |
| Wang | No steroids. In addition, all the patients were required to rest remaining in bed and avoiding lifting the affected limb, and avoid overtiring, tension and cold; were given a low purine diet, abstaining from smoking, wine, animal giblets, fish, shrimp and unfermented soybean foods; were required to drink enough water, 2000-3000 ml daily; received sodium bicarbonate 1.0 g three times daily orally to alkalinize the urine; received etoricoxib 60 mg daily orally for 10 days to relieve pain in the affected joints |
| Wei | NSAIDs were part of the control group. Allopurinol and colchicine are not mentioned in the study |
| Xie | At baseline, every patient was instructed to reduce their purine- rich food intake and to drink >2 L of water a day. Exclusion criteria included administration of uricosuric medication within 2wks. Treatment with other gout therapies during the study period rendered the participant as an invalid case. |
| Yu | If the patient had already received treatment for gout, he/she had to undergo a two-week washout period; only those whose sUA remained >420 μmol/L after the washout period could be included. Patients were excluded if they were taking concurrent hypouricemic medications. All patients were advised not to consume purine-rich diets. |

CS: corticosteroids; NSAIDs: non-steroidal anti-inflammatory drugs; SUA: serum uric acid.

*Supplementary table 9. Inclusion and exclusion criteria of studies regarding diets*

| First author | Inclusion and exclusion criteria |
| --- | --- |
| Christensen | >18 yr old; obesity (BMI > 30); 2015 EULAR/ACR criteria; SU level of at least 5.0 mg/dL; at least one self-reported gout flare in the past year. Exclusion criteria: Other inflammatory diseases; cancer; took part in other trials, including pharmacological studies or weight loss trials |
| Holland | >18 yr old; history of gout as per ACR criteria; on a stable dose of urate lowering therapy at target (SU<0.36 mmol/L). Exclusion criteria: unable to communicate in English (both verbal and written, to standardize information) |
| Juraschek | Inclusion criteria: ≥18 yr, self- reported diagnosis of gout; SU concentration ≥7 mg/dL; Exclusion criteria: active use of or plans for urate lowering therapy, excessive alcohol use, stage 4 or 5 chronic kidney disease, unstable medication use (steroid, lipid-lowering, or antihypertensive agents), active prescriptions of warfarin or insulin, major gastrointestinal conditions affecting food absorption, or inability to store food at home. |
| Kretova | Gout, hyperuricemia (males ≥0,42 mmol/L and females ≥0,36 mmol/L), abdominal obesity (waist circumference of ≥102 cm for males and ≥88 cm for females), and not receiving urate-lowering therapy. Exclusion criteria : Concurrent presence of other forms of inflammatory joint disease than gout; intractable gout due to side effects or contraindications for standard flare treatment (NSAIDs, colchicine and corticosteroids); current use of urate lowering therapy or use of urate lowering therapy in the last 30 days; indication for urate lowering therapy according national guidelines (NVR gout 2013) including 2 or more flares in one year, tophaceous gout or history of urate urolithiasis unless agreement between patient and treating rheumatologist led to the decision to postpone the start of ULT for the duration of at least the first 4 months of the study; pregnancy; insufficient comprehension of Dutch language; already following a (near-)vegan diet; in case of smoking, unwillingness to stop smoking for at least the duration of the study; low e-health competencies (lowest proficiency according to Pharos quick scan); inability to be scheduled for counselling and measurement visits; psychiatric disease; no informed consent |

ACR: American College of Rheumatology; BMI: body mass index (kg/m^2^); EULAR: European Alliance of Associations for Rheumatology; kg: kilogram; m:meter; ULT: urate-lowering therapy.

*Supplementary table 10. Concurrent gout treatment of studies regarding diet*

| First author | Gout treatment and anti-inflammatory/urate lowering medication |
| --- | --- |
| Christensen | Participants were allowed to use ULT, but they were not allowed to change ULT type or dose during the trial |
| Holland | Stable dose of urate lowering therapy at target (serum urate <0.36 mmol/L), |
| Juraschek | Exclusion criteria included active use of or plans for urate lowering therapy, unstable medication use (steroids). Colchicine and NSAIDS in both groups were used |
| Kretova | Participants were allowed to use their usual gout flare medication during a gout flare |

NSAIDs: non-steroidal anti-inflammatory drugs; ULT: urate-lowering therapy.
